# The oncogenic lncRNA *MIR503HG* suppresses cellular senescence counteracting supraphysiological androgen treatment in prostate cancer

**DOI:** 10.1186/s13046-024-03233-2

**Published:** 2024-12-16

**Authors:** Julia Kallenbach, Mahdi Rasa, Mehdi Heidari Horestani, Golnaz Atri Roozbahani, Katrin Schindler, Aria Baniahmad

**Affiliations:** 1 Institute of Human Genetics, Jena University Hospital, Am Klinikum 1, Jena, 07740 Germany; 2 Leibniz Institute on Aging - Fritz Lipmann Institute (FLI), Jena, Germany; 3 Institute of Immunology, University Hospital, Kiel, Schleswig-Holstein Germany

**Keywords:** Prostate cancer, Androgen receptor, Supraphysiological androgen level, Bipolar androgen therapy, Long non-coding RNAs, Cellular senescence

## Abstract

**Background:**

The androgen receptor (AR), a ligand-dependent transcription factor, plays a key role in regulating prostate cancer (PCa) growth. The novel bipolar androgen therapy (BAT) uses supraphysiological androgen levels (SAL) that suppresses growth of PCa cells and induces cellular senescence functioning as a tumor suppressive mechanism. The role of long non-coding RNAs (lncRNAs) in the regulation of SAL-mediated senescence remains unclear. This study focuses on the SAL-repressed lncRNA *MIR503HG*, examining its involvement in androgen-controlled cellular senescence in PCa.

**Methods:**

Transcriptome and ChIP-Seq analyses of PCa cells treated with SAL were conducted to identify SAL-downregulated lncRNAs. Expression levels of *MIR503HG* were analyzed in 691 PCa patient tumor samples, mouse xenograft tumors and treated patient-derived xenografts. Knockdown and overexpression experiments were performed to assess the role of *MIR503HG* in cellular senescence and proliferation using senescence-associated β-Gal assays, qRT-PCRs, and Western blotting. The activity of *MIR503HG* was confirmed in PCa tumor spheroids.

**Results:**

A large patient cohort analysis shows that *MIR503HG* is overexpressed in metastatic PCa and is associated with reduced patient survival, indicating its potential oncogenic role. Notably, SAL treatment suppresses *MIR503HG* expression across four different PCa cell lines and patient-derived xenografts but interestingly not in the senescence-resistant LNCaP Abl EnzaR cells. Functional assays reveal that *MIR503HG* promotes PCa cell proliferation and inhibits SAL-mediated cellular senescence, partly through miR-424-5p. Mechanistic analyses and rescue experiments indicate that *MIR503HG* regulates the AKT-p70S6K and the p15^INK4b^-pRb pathway. Reduced expression of *MIR503HG* by SAL or knockdown resulted in decreased *BRCA2* levels suggesting a role in DNA repair mechanisms and potential implications for PARP inhibitor sensitivity by SAL used in BAT clinical trial.

**Conclusions:**

The lncRNA *MIR503HG* acts as an oncogenic regulator in PCa by repressing cellular senescence. SAL-induced suppression of *MIR503HG* enhances the tumor-suppressive effects of AR signaling, suggesting that *MIR503HG* could serve as a biomarker for BAT responsiveness and as a target for combination therapies with PARP inhibitors.

**Supplementary Information:**

The online version contains supplementary material available at 10.1186/s13046-024-03233-2.

## Background

Prostate cancer (PCa) constitutes to the most commonly diagnosed cancers and ranks as the second leading cause of cancer-related mortality among men in Western countries [1]. The androgen receptor (AR), a transcription factor activated by androgens, regulates PCa-related processes such as proliferation, differentiation and the prostate specific gene expression [2, 3]. Hence, therapeutic approaches to suppress AR-mediated signaling including androgen deprivation therapy and AR antagonists are the main hormone-based strategies to inhibit PCa growth [4, 5]. However, most patients will gradually develop resistance against these therapies leading to progression of the cancer from a castration-sensitive (CSPC) to a castration-resistant stage (CRPC), where cancer grows despite low androgen level [6].

Intriguingly, paradoxical observations demonstrated that besides androgen deprivation, also supraphysiological androgen level (SAL) suppresses the PCa growth in different preclinical models including CSPC and CRPC cell lines as well as xenograft models [7–11]. These findings have led to the development of an innovative therapy for patients with metastatic PCa, termed bipolar androgen therapy (BAT), which involves sequential cycling between supraphysiologic and near-castrate serum testosterone level [12–14]. The alternation between very high and low testosterone level intends to block adaptive changes of the AR signaling and to resensitize the cancer to antiandrogen therapy [14–16]. BAT is currently evaluated in phase II clinical trials, including TRANSFORMER, RESTORE and COMBAT, for patients with metastatic CRPC [14, 17, 18].

Previously, we demonstrated that SAL induces cellular senescence, a state of growth arrest that limits cancer growth, in several PCa model systems including 2D and 3D spheroid cell culture, mouse xenograft tumors and ex vivo treated patient-derived PCa tissue [7, 10, 11]. The exact mechanisms and pathways underlying the tumor-suppressive activity of AR leading to growth suppression and cellular senescence are not fully understood. The SAL-mediated senescence is regulated by the p15^INK4b^-pRb-E2F1 pathway [7]. Moreover, SAL enhances the phosphorylation of AKT and activates the pro-survival AKT signaling, which mediates in part the androgen-induced cellular senescence [11].

Long non-coding RNAs (lncRNAs) are a diverse class of RNA molecules longer than 200 nucleotides, that lack protein-coding potential but play crucial regulatory roles in various biological processes [19]. It has been reported that lncRNAs are part of competing endogenous RNA (ceRNA) networks regulating tumorigenesis by influencing cell proliferation, apoptosis, migration, metastasis, and the metabolic reprogramming of cancer cells functioning as tumor suppressors or oncogenes [20, 21].

Recent studies have shown that lncRNAs are emerging as important regulators of cellular senescence [22, 23]. However, the functions and regulations of numerous lncRNAs in the process of SAL-induced cellular senescence are poorly understood.

In this study, we identified the MIR503 host gene (*MIR503HG*) as a novel SAL-downregulated lncRNA through transcriptome analysis of our RNA-Seq datasets from both castration-sensitive and castration-resistant PCa cell lines [11, 24]. The lncRNA *MIR503HG* is located on chromosome Xq26.3 and has been reported as a tumor suppressor gene in various human cancers including hepatocellular carcinoma, gastric cancer, colon cancer, and ovarian cancer [25–28]. However, the role of *MIR503HG* in PCa and SAL-mediated cellular senescence remains to be elucidated.

Here, we analyzed an extensive dataset comprising 691 tissue samples. This large cohort contains 52 normal tissue samples, 539 primary tumor, and 100 metastatic samples obtained from TCGA. We found that *MIR503HG* expression is upregulated in metastatic PCa tumor compared to primary tumor and normal tissue and is correlating with poor survival of patients with prostate adenocarcinoma suggesting an oncogenic function of *MIR503HG*. Functional assays revealed that *MIR503HG* promotes the growth of PCa cell lines and 3D tumor spheroids and inhibits the induction of the SAL-mediated cellular senescence indicating rather an oncogenic role of *MIR503HG* in PCa. Mechanistically, *MIR503HG* inhibits cell senescence in part through targeting miR-424-5p leading to the suppression of the p15^INK4b^-pRb-E2F1 pathway. A currently ongoing clinical trial uses the combination of BAT with the PARP inhibitor Olaparib with good clinical response of patients [29]. Interestingly, in line with its oncogenic activity, *MIR503HG* upregulates the SAL-repressed homology-directed DNA repair gene *BRCA2.* Since SAL represses the expression of the lncRNA *MIR503HG* it may support mechanistically the use of PARP inhibitors and suggests that *MIR503HG* is involved in homology-directed DNA repair in PCa cells.

## Materials and methods

### Correlation analysis

The expression data from 52 normal prostate tissue, 539 primary tumors, and 100 metastatic tumors, was downloaded from TCGA (The Cancer Genome Atlas) database. FPKM_uq_unstranded (Fragment Per Kilobase pair transcript per million sequenced reads, uniquely mapped, both DNA strands) was used for downstream analysis. All the analyses were performed using R (v4.2.3). The correlation coefficient of the expression of each gene compared to *MIR503HG* was calculated using cor.test with method = “pearson” parameters. Genes with a correlation coefficient > 0.5 were used for the downstream analysis. For gene ontology (GO) analysis, genes were mapped to the identifier GO term using annFUN.org function (topGO v2.50.0) with whichOnto="BP”, feasibleGenes = NULL, mapping="org.Hs.eg.db” parameters. Subsequently, topGOdata object was created using the new (method package from R base) function with class="topGOdata”, ontology="BP”, annot = annFUN.GO2genes, nodeSize = 10 parameters. The enrichment p-value was calculated using the Fisher exact test by runTest function (topGO) with algorithm = “elim”, statistic = “fisher” parameters. Using GenTable function (topGO), the summary for the enrichment analysis was created.

### Cell culture and treatment

The castration-sensitive, androgen-dependent LNCaP (lymph node carcinoma of the prostate) and castration-resistant androgen-independent C4-2 cell lines were cultured as described previously [7]. LNCaP Abl EnzaR cells were obtained from Hoefer et al. [30] and cultured in RPMI 1640 Medium GlutaMAX (Thermo Fisher Scientific, Waltham, Massachusetts) containing 10% double charcoal stripped FBS and 1% penicillin/streptomycin. Cells were treated with 0.1% DMSO as solvent control, 1 nM R1881 at SAL and/or with 3 µM LY2584702 (Selleckchem) [7].

### Cell transfection

To investigate the function of *MIR503HG* in PCa cells, *MIR503HG* was either silenced using small interfering RNA (siRNA) or overexpressed. Human *MIR503HG* siRNA (si*MIR503HG*) and non-targeting Control (siControl) siRNA (Supplemental Table S1) were purchased from Dharmacon. Transient transfection was performed using DharmaFECT reagent 3 according to the manufacturer’s instructions (Horizon Discovery, Cambridge, UK). Transfection experiments were conducted 24 h prior treatment of cells.

The full-length sequence of *MIR503HG* was purchased from Eurofins Genomics (Ebersberg, Germany) and inserted into the pCDH-CMV vector. The constructed vector was transfected into PCa cells using jetPRIME DNA and siRNA transfection reagent from Polyplus (Sartorius, Göttingen, Germany) based on the manufacturer’s guidelines.

The miR-424-5p inhibitor (5’-CAGCAGCAAUUCAUGUUUUGAA-3’), miR-424-5p mimic (5’-CAGCAGCAAUUCAUGUUUUGAA-3’) and microRNA mimic negative control were obtained from Dharmacon and transfected with DharmaFECT reagent.

### Senescence-associated beta-galactosidase (SA β-Gal) staining

Cellular senescence was analyzed by senescence-associated beta-galactosidase (SA β-Gal) activity staining as described elsewhere [7]. The percentage of SA β-Gal positive cells was calculated by counting three areas with at least 200 cells per well under a light microscope (Zeiss, Oberkochen, Germany).

### Cell viability assay

Cell viability of LNCaP and C4-2 cells was examined by crystal violet (CV) staining. The absorbance at 590 nm was measured with an UV/Vis spectrometer SPECORD 50 (Analytic Jena, Jena, Germany) as indirect measure of growth.

### Reverse-transcription quantitative real-time PCR (qRT-PCR)

RNA from cells was extracted using RNA-Solv Reagent (VWR, Darmstadt, Germany) based on manufacturer’s instructions. cDNA synthesis was carried out using the High Capacity cDNA Reverse Transcription kit (Applied Biosystems, Foster City, CA, USA). qRT-PCR was conducted using SsoAdvanced Universal SYBR Green Supermix (Bio-Rad, Munich, Germany) and the Bio-Rad CFX96 Real Time PCR detection system. The primer sequences are listed in Supplemental Table S2. Gene expression was normalized to the house-keeping genes *TBP* and *α-Tubulin*.

### Protein extraction and Western blotting

Cells were harvested and lysed in NETN buffer (20 mM Tris-HCl pH 8.0, 100 mM NaCl, 1 mM EDTA, 1% Tergitol (NP-40), 50 mM NaF, 100 µM Na_3_VO_4_, 10 mM β-glycerophosphate). Protein concentrations were quantified by BCA assays (Thermo Fisher Scientific). Protein lysates were separated by 12% or 15% SDS-PAGE and proteins were transferred to PVDF membranes. The membranes were blocked using skim milk and then incubated with primary antibodies. Horseradish peroxidase-conjugated anti-mouse IgG or anti-rabbit IgG were used as secondary antibodies. Protein levels were detected by ImageQuant™ LAS 4000 (GE Healthcare Bio-Sciences AB, Germany). The bands were quantified with the LabImage D1 software. All antibodies are listed in Supplemental Table S3.

### 3D tumor spheroid generation and immunofluorescence staining

Spheroids of knockdown and control transfected cells were formed using the previously described forced floating method [31]. Spheroids were treated for 6 days with SAL or DMSO (10 technical replicates each) followed by fixation with 4% PFA. Subsequently, SA β-Gal activity staining and Ki67 immunofluorescence staining were conducted according to the previously described protocol [10].

### Chromatin immunoprecipitation sequencing (ChIP-Seq)

Chromatin immunoprecipitation of AR was performed according to the manufacturer’s protocol (iDeal ChIP-seq Kit Diagenode, Cat.-Nr.: C01010055, Denville, U.S.). The antibodies used for ChIP are listed in Supplement Table S3. ChIP-Seq library preparation (TruSeq ChIP-seq) and ChIP-sequencing were performed by Macrogen (Seoul, South Korea) using the NovaSeq 6000 platform with an output of 5 Gb (30 Mio reads) per sample.

The fastq quality was assessed using FastQC (v0.12.1). The adaptor and low-quality sequences were removed using trim_galore (v0.6.10) with default parameters. The data was mapped to the reference genome (GRch37/hg19) with bowtie (v1.1.2, Ben Langmead, College Park, MD, USA) using --best --strata -m 1 -n 2 parameters. Duplicated reads were removed using a customer script. The peak calling was performed using macs14 (v1.4.2) with --gsize = hs -p 1e-3 parameters and the input sample as the control. To define the reference region, the 1e-3 peaks of all the replicates were pooled using peakreference R function (TCseq package v1.22.6). After defining the reference region from 1e-3 peaks, the read count was calculated for each region in all the replicates (a custom script), and p-value was calculated using DESeq function (DESeq2 v1.38.3). The peaks with adjusted p-value < 0.05 were considered as differentially regulated regions. Motif enrichment analysis was done using findMotifsGenome.pl script (Homer v4.9.1) with genome = hg19 -len 4,6,8,10,12 -size given parameters.

### Mouse xenograft study

Patient-derived xenografts (PDX) datasets from GSE188176 were utilized. Immunocompromised, non-castrated nude mice (8 weeks old, Janvier Labs, France) were subcutaneously (s.c.) injected into both flanks with C4-2 cells (10^6^ cells per 50 µl 1xPBS) mixed 1:1 with Matrigel (CORNING; USA; 356231). Mice with a tumor size of approximately 80 mm^3^ were daily s.c. injected with vehicle (0.5% Tween 80) or dihydrotestosterone (AbMol BioScience; USA; M6033) corresponding to SAL (50 mg/kg). Mice were weighted every other day. Tumor size was measured with a caliper, and mice were sacrificed either when tumor reached a size of approximately 800 mm^3^, when mice showed weight loss more than 20% of the initial weight, or after 5 weeks of injection. RNA was extracted from frozen tumors according to the previously described protocol [11]. Data were used without outliers. The animal experiments were approved by the Thüringer Landesamt für Lebensmittelsicherheit und Verbraucherschutz, Germany (Reg.-Nr.: UKJ-23-013).

### *Ex vivo* treatment of prostatectomy samples

Patient-derived prostatectomy tissue was sectioned into 3 × 3 mm sections and cultured at 5% CO_2_ and 37 °C for 48 h in RPMI supplemented with 5% FCS, 1% penicillin/streptomycin, 1% sodium pyruvate and 25 mM HEPES (pH 7.5), 1 µM R1881 (SAL) or 0.1% DMSO as control. The tissue samples were frozen in 1 ml TriFast™ per 100 mg tissue weight. The homogenization of the tissues was achieved with the QIAGEN TissueLyser LT using Quiagen steel beads (5 mm) for 9 min at the frequency of 50 Hz. After an incubation of 10 min at room temperature the TriFast™-tissue mixture was centrifuged at 12,000 g, 4 °C for 10 min. RNA extraction was further performed as described previously. All patients provided informed consent and were informed about the purpose of the study [7]. The study was approved by the Ethics Committee of the Friedrich-Schiller-University (2019–1502-Material), in accordance with the Declaration of Helsinki. The *MIR503HG* levels were measured in patient samples with Gleason score of 6–7, with presurgery PSA values ranging from 7 to 28 ng/ml.

### Statistical analysis

The data were statistically analyzed using GraphPad Prism 8.0 software (RRID: SCR_000306). To compare the mean values between two groups, a two-tailed unpaired *t*-test was performed and for multiple comparisons two-way ANOVA was used. Mean, standard deviation (SD), and standard error of mean (SEM) were calculated from three biological replicates.

## Results

### Identification of the SAL-downregulated lncRNA *MIR503HG* as a novel factor in AR signaling impacting the survival outcome of prostate adenocarcinoma (PRAD) patients

SAL suppresses the growth of PCa cells in vitro and seems to be beneficial in clinical trials treating patients within the BAT [7, 9, 15]. Intriguingly, SAL induces cellular senescence in CSPC and CRPC cells as well as in patient prostatectomy specimens treated ex vivo with SAL and in CRPC xenograft model [7, 10]. However, the exact underlying pathways and molecular networks implicated in the growth suppression and induction of cellular senescence by SAL remain largely unknown. Since the role of lncRNAs in the regulation of cellular senescence is poorly understood, we aimed to identify novel lncRNAs involved in the SAL-mediated cellular senescence. To date, transcriptome analyses have predominantly examined potential protein coding tumor suppressor genes upregulated by SAL treatment [32]. In this study, we focused on potential lncRNA suppressors of the SAL-mediated cellular senescence, particularly targeting oncogenic lncRNAs that are downregulated by SAL treatment. To examine differentially downregulated ncRNAs associated with SAL-induced cellular senescence in PCa cells, we analyzed our RNA-Seq transcriptome data (GSE155528, GSE172205) of both, the castration-sensitive LNCaP and castration-resistant C4-2 cell lines, treated with 1 nM of the synthetic androgen R1881 at SAL compared to DMSO as solvent control.

**Fig. 1:**
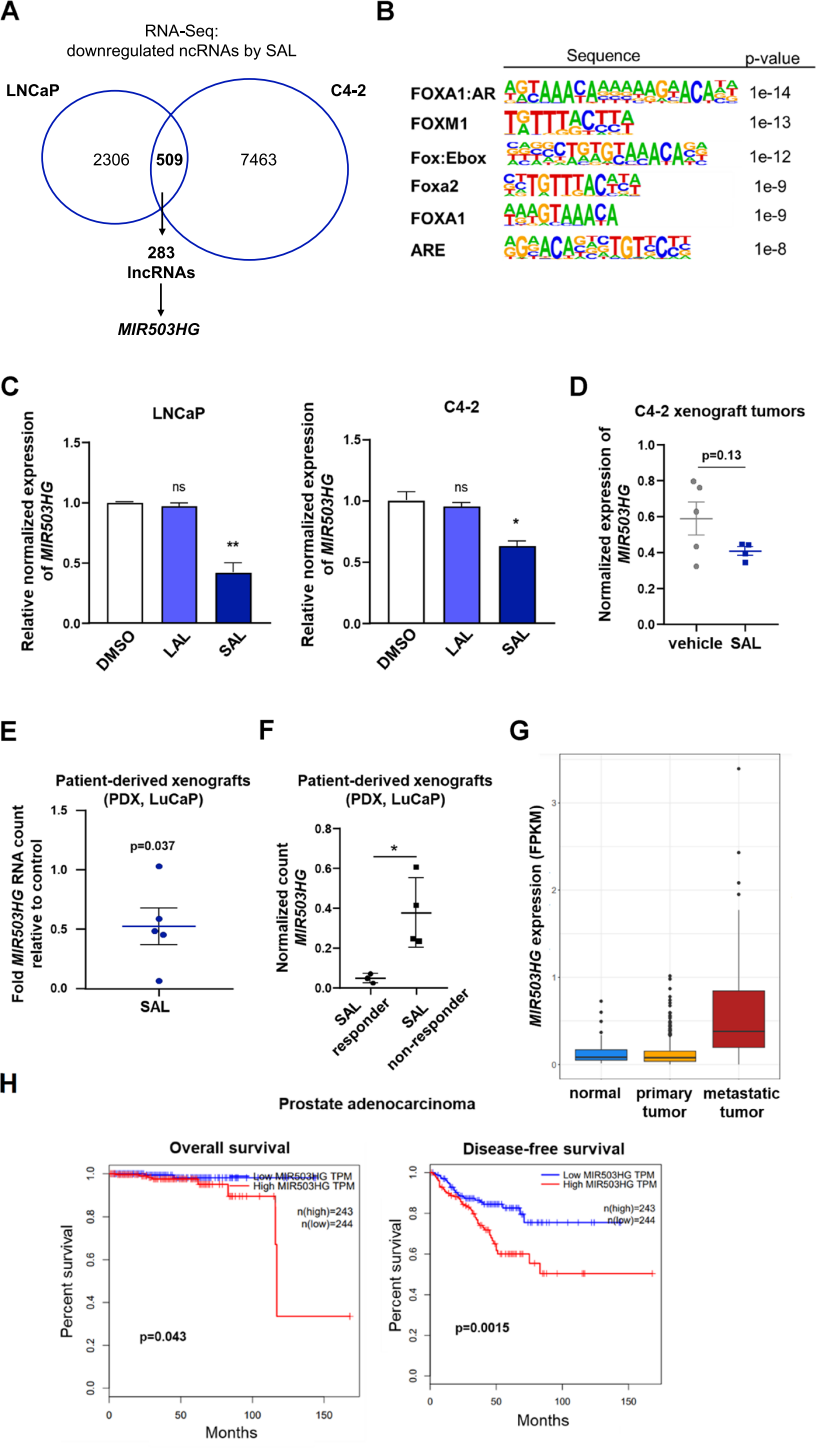
LncRNA *MIR503HG* expression is downregulated by SAL in PCa cell lines, in mouse xenografts and patient-derived xenografts (PDX). LNCaP and C4-2 cells were treated with LAL (1 pM R1881), SAL (1 nM R1881) or DMSO as solvent control for 72 h. **A** Venn diagram indicates the number of ncRNAs downregulated by SAL treatment in both cell lines using RNA-Seq data. **B** Transcription factor motifs of the AR binding sites at androgen-repressed ncRNA genes. **C** *MIR503HG* mRNA level in treated LNCaP and C4-2 cells were detected by qRT-PCR normalized to *TBP* and *α-Tubulin*. Data represent the mean ± SEM (*n* = 3). One-tailed unpaired Student’s *t* -test was performed for statistical analysis compared to solvent control (**p ≤ 0.01, ***p ≤ 0.001, ns: not significant). **D** *MIR503HG* mRNA expression of C4-2 xenograft tumors treated with DHT at SAL (*n* = 4) compared to vehicle (*n* = 5) was analyzed by qRT-PCR. Data represent the mean ± SEM. **E** Patient-derived xenograft (PDX) tumors (*n* = 5, LuCaP) were analyzed for normalized *MIR503HG* RNA count of supraphysiological testosterone treated PDX LuCaP tumors (*n* = 5) relative to control treatment (set as 1). Data represent the mean  ±  SEM. **F** Normalized RNA count of *MIR503HG* from SAL-treated PDX tumor responders (*n* = 3) and non-responders (*n* = 4). **G** TCGA samples were used to analyze the expression of *MIR503HG* in normal tissue (*n* = 52), primary (*n* = 539) and metastatic PCa (*n* = 100) tumors. **H** Overall survival and disease-free survival curve of prostate adenocarcinoma (PRAD) patients with high *MIR503HG* (red curve; n  = 243) or low *MIR503HG* (blue curve; *n* = 244) expression analyzed by GEPIA

 Global transcriptome analysis revealed 509 commonly downregulated ncRNAs in both cell lines, among which 283 were lncRNAs (Fig. 1A). Notably, our ChIP-Seq of AR indicated a recruitment to 251 ncRNAs. Motif analysis of the AR binding sites within these downregulated ncRNAs identified motifs for FOXA1:AR and FOXM1 (Fig. 1B) suggesting that these pioneering factors are also involved in androgen-mediated repression. Among the SAL-downregulated lncRNAs, we identified *MIR503HG*, a lncRNA aberrantly expressed in different cancer types [33]. However, the role of the lncRNA *MIR503HG* in PCa and hormonal control remains unexplored.

To confirm the androgen regulation of *MIR503HG* expression level qRT-PCRs were performed for LNCaP and C4-2 cells treated with either SAL (1 nM), low androgen level (LAL, 1 pM) of R1881, or DMSO as control. SAL treatment significantly decreased the expression of *MIR503HG* in LNCaP cells, whereas LAL treatment showed no significant change in the expression level of the lncRNA (Fig. 1C) suggesting an androgen concentration-dependent transcriptional regulation and repression of *MIR503HG* specifically by SAL. Similar effects have been observed in C4-2 cells (Fig. 1C). The analysis of other PCa cell lines revealed, that the gene expression of *MIR503HG* was significantly reduced by SAL in both VCaP cells (Fig. S1A) and LAPC4 cells (Fig. S1B). Further the *MIR503HG* expression in C4-2 xenograft tumor samples from mice treated either with dihydrotestosterone (DHT) at SAL or vehicle was analyzed and confirmed the downregulation of *MIR503HG* by SAL, although this effect was not statistically significant (Fig. 1D). Evaluation of the *MIR503HG* gene expression in prostatectomy specimens treated ex vivo with SAL for 48 h revealed a reduction in mRNA levels by qRT-PCR (Fig. S1C), however the reduction did not reach statistical significance, which could be explained by the heterogeneity of the native tissue. Since both SAL and Enzalutamide (Enza) induced senescence in LNCaP cells [11, 34] but interestingly not in the castration resistant and Enza resistant LNCaP Abl EnzaR cells (Fig. S1D), we examined the mRNA expression of *MIR503HG* in these cells. In line with the resistance to senescence induction, SAL or Enza treatment did not measurably affect mRNA level of the cell cycle regulators *E2F1* and *CCND1* in LNCaP Abl EnzaR cells (Fig. S1E, F). Notably, and in line with the lack of senescence induction, SAL treatment failed to repress *MIR503HG* expression in these cells (Fig. S1G). Whereas Enza did not significantly reduce *MIR503HG* expression in LNCaP cells, it rather significantly upregulated the expression of this lncRNA in LNCaP Abl EnzaR cells (Fig. S1G). The data suggest that the repression of the lncRNA *MIR503HG* by SAL is associated with cellular senescence.

Noteworthy, a significant decrease of *MIR503HG* RNA counts (Fig. 1E) was detected in LuCaP patient-derived xenografts (PDX) treated with supraphysiological testosterone using published RNA-Seq data (GSE188176) [35]. Intriguingly, SAL-responding tumors exhibited significantly lower *MIR503HG* expression compared to non-responder tumors (Fig. 1F) indicating beneficial effects of low *MIR503HG* expression and a potential role of MIR503HG as a predictive biomarker for the treatment response.

Additionally, we analyzed a larger number of 691 RNA-Seq samples from normal prostate tissue, primary PCa and metastatic tumor obtained from TCGA. Interestingly, we observed that *MIR503HG* was significantly overexpressed in metastatic PCa tumors compared to normal tissue and primary tumors (Fig. 1G). This observation suggests that this lncRNA might have an oncogenic function and is involved in tumor progression. To assess the clinical relevance of these findings, the survival of patients with prostate adenocarcinoma (PRAD) was analyzed utilizing Gene Expression Profiling Interactive Analysis (GEPIA). The data revealed that both, the overall and disease-free survival of patients with PRAD were significantly decreased in patients with high *MIR503HG* expression (Fig. 1H) indicating that lower *MIR503HG* expression is beneficial for survival and further supports the notion of an oncogenic role of *MIR503HG* in PCa.

Further, we performed correlation analysis using the TCGA datasets. Notably, the expression of 724 genes was positively correlated with *MIR503HG* expression (using the correlation coefficient > 0.5). We observed a positive correlation between *MIR503HG* and *CDKN2B-AS1* (ANRIL) (Supplemental Table S5), a lncRNA inhibiting cellular senescence through negative regulation of *CDKN2A* and *CDKN2B* expression, which encode the important cell cycle inhibitors p16^INK1A^ and p15 ^INK1A^, respectively [36]. Both cell cycle inhibitors mediate the SAL-induced cellular senescence [7, 11].

Interestingly, GO pathway enrichment analysis of the gene set positively correlated with *MIR503HG* expression indicated positive regulation of critical pro-tumorigenic pathways including angiogenesis, endothelial cell migration and epithelial cell proliferation (Table 1), while anti-tumorigenic pathways such as endothelial cell apoptotic process and cell adhesion are indicated to be negatively regulated (Supplemental Table S4). The analysis further supports the notion that the lncRNA *MIR503HG* is involved in PCa progression.

Collectively, the data indicate that the *MIR503HG* is part of AR signaling in PCa. SAL treatment reduces *MIR503HG* expression in different PCa cell lines in vitro as well as in mouse CRPC xenografts. Further, the analyses indicate an oncogenic activity of lncRNA *MIR503HG*, suggesting a potential tumor-suppressive activity by SAL.

**Table 1:** GO pathways enriched for genes positively correlated with *MIR503HG* expression

Pathways	*p*-value
Positive regulation of angiogenesis	0.00043
Positive regulation of mitotic cell cycle spindle assembly checkpoint	0.00185
Endothelial cell migration	0.00295
Positive regulation of cell migration	0.01004
Negative regulation of endothelial cell apoptotic process	0.01536
Negative regulation of cell adhesion	0.02264
Nositive regulation of epithelial cell proliferation	0.0307

Correlation analysis was conducted with 52 normal tissue samples, 539 primary and 100 metastatic PCa tumor samples from TCGA database (genes with correlation coefficient of > 0.5 were used)

### Knockdown of *MIR503HG* suppresses growth of castration-sensitive and castration-resistant PCa cells by induction of cellular senescence

**Fig. 2:**
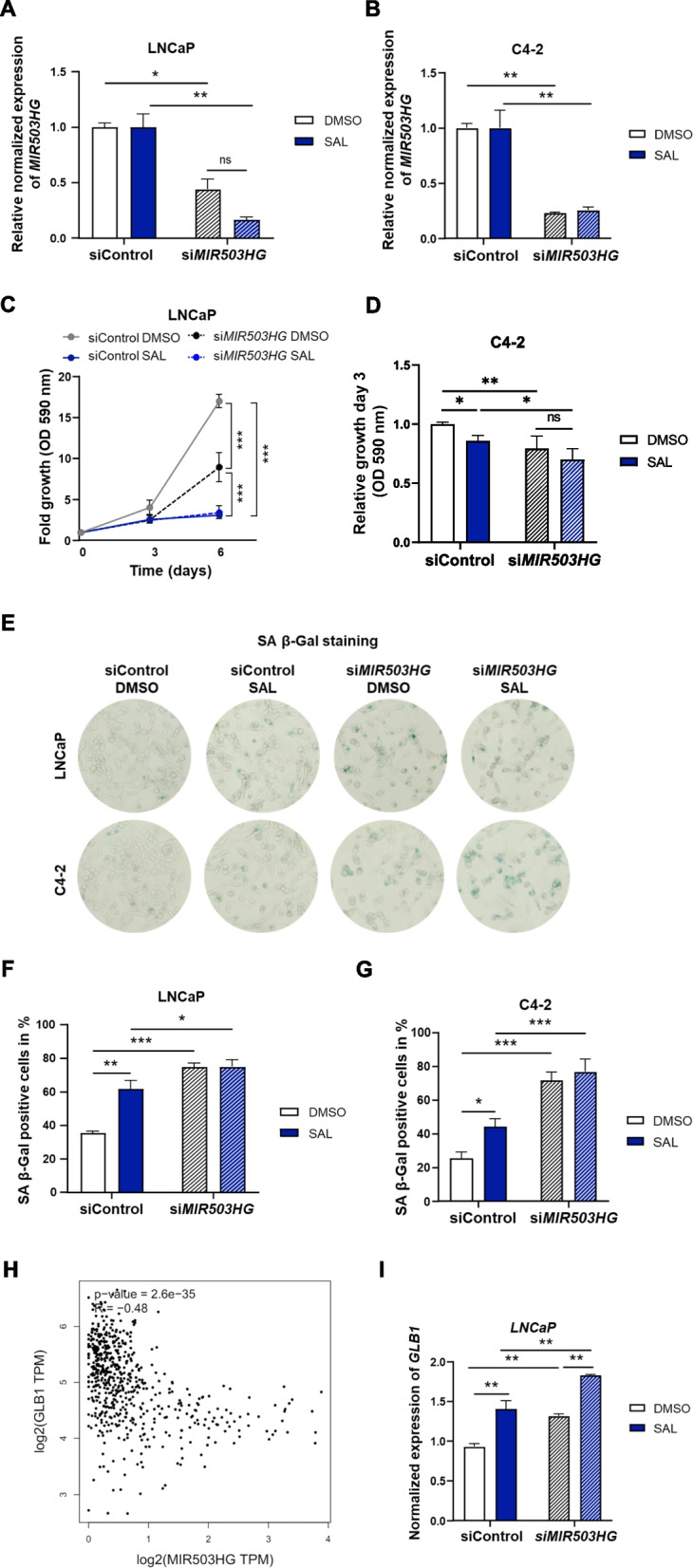
Knockdown of *MIR503HG* suppresses growth of androgen-sensitive and castration-resistant PCa cells and induces cellular senescence. LNCaP and C4-2 cells were transfected with siRNA targeting *MIR503HG* (si*MIR503HG*) or non-targeting siRNA (siControl) and treated with DMSO or SAL. **A**, **B** Expression of *MIR503HG* was measured by qRT-PCR in *MIR503HG* depleted cells relative to control transfected cells for each treatment. Bar graphs represent mean ± SEM from two independent experiments with four technical replicates. **C** Growth curve of si*MIR503HG* transfected LNCaP cells treated for 6 days. **D** Growth of *MIR503HG* depleted C4-2 cells following 72 h SAL treatment. Two-way ANOVA was performed for multiple comparisons (*n* = 3) (*p ≤ 0.05, **p ≤ 0.01, ***p ≤ 0.001, ns: not significant). **E** Representative pictures of SA β-Gal staining. **F**, **G** The percentage of the SA β-Gal positive LNCaP (F) and C4-2 (**G**) cells was calculated in relation to the total cell counts. Bar graphs are shown as mean ± SD (*n* = 3). **H** Correlation analysis of *GLB1* and *MIR503HG* expression using TCGA PRAD and GTEx prostate datasets from GEPIA. Spearman correlation coefficient was calculated (*R* = −0.48, *p*-value = 2.6e-35). **I** Expression of *GLB1* in LNCaP cells transfected with si*MIR503HG* or siControl and treated with SAL or DMSO normalized to *TBP* and *α-Tubulin*. Data represent the mean ± SEM

To investigate whether *MIR503HG* is involved in PCa progression by regulating SAL-mediated cellular senescence, knockdown experiments using siRNA were performed to analyze the impact of *MIR503HG* on the proliferation of PCa cells. Transfection of LNCaP and C4-2 cells with si*MIR503HG* efficiently silenced expression of *MIR503HG* after treatment with SAL or DMSO in both cell lines (Fig. 2A and B). The knockdown of *MIR503HG* potently suppressed growth of the androgen-sensitive LNCaP (Fig. 2C, Fig. S2) and castration-resistant C4-2 cells (Fig. 2D, Fig. S2) in DMSO treated cells. Notably, treatment with SAL further inhibits the growth of both LNCaP and C4-2 cells by *MIR503HG* knockdown. These data support the hypothesis that *MIR503HG* promotes PCa growth.

Interestingly, analysis of the SA β-Gal activity showed a strong increase of senescent cells following *MIR503HG* knockdown in LNCaP (Fig. 2E, F) and C4-2 cells (Fig. 2E, G), indicating that *MIR503HG* functions as critical suppressor of SAL-induced cellular senescence in PCa cells. Consistent with the repression of cellular senescence by *MIR503HG*, expression of *MIR503HG* is negatively correlated with the *GLB1* expression, which encodes the senescence-associated beta-galactosidase 1, an enzyme with increased activity in senescent cells catalyzing the hydrolysis of beta-galactoside (Fig. 2H). Depletion of *MIR503HG* increased mRNA level of *GLB1* in LNCaP cells (Fig. 2I), confirming the negative correlation observed in the TCGA PRAD datasets.

These findings highlight the role of *MIR503HG* in androgen signaling to promote PCa cell proliferation, and to suppress cellular senescence, implicating *MIR503HG* as an oncogenic lncRNA and as a potential therapeutic target for PCa.

### *MIR503HG* depletion suppresses growth of 3D PCa tumor spheroids

**Fig. 3:**
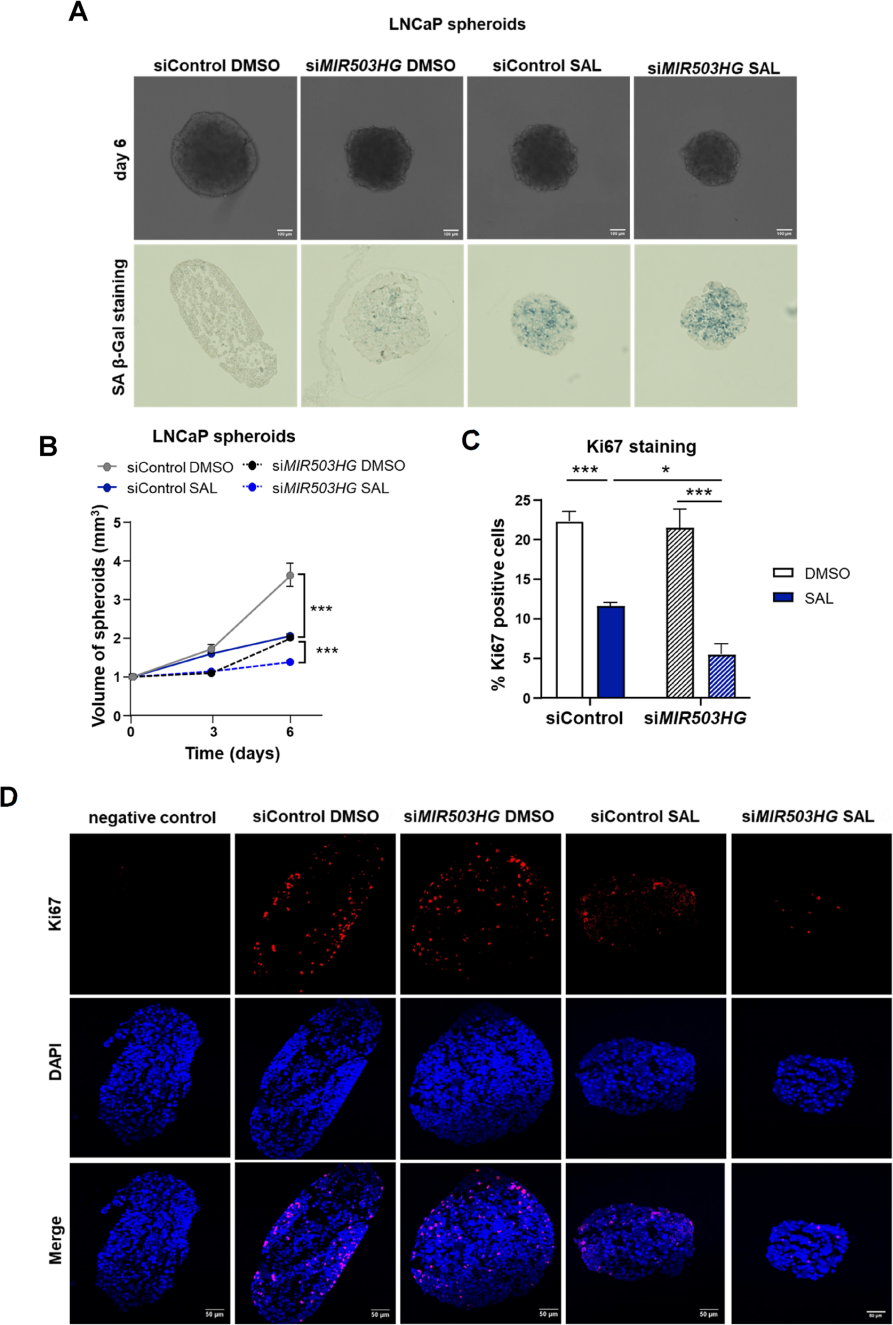
*MIR503HG* depletion inhibits growth of PCa tumor spheroids. LNCaP spheroids of siControl and si*MIR503HG* transfected cells were generated and treated with SAL or DMSO for 6 days. **A** Representative size of LNCaP tumor spheroids and senescence-associated beta-galactosidase (SA β-Gal) staining as a marker of cellular senescence. Scale bars correspond to 100 μm. **B** Growth curve showing the volume of the spheroids. The mean ± SEM values are represented (*n *= 2, each contains ten technical replicates). Two-way ANOVA was performed for statistical analysis. **C** Immunofluorescence staining detects the proliferation marker Ki67 (red), DAPI (blue), and merge. Samples without primary antibody serve as a negative control. Scale bars correspond to 50 μm. **D** Ki67 positive cells were quantified by ImageJ software (*p ≤ 0.05, ***p ≤ 0.001)

Next, the effect of *MIR503HG* on the proliferation of three-dimensional (3D) spheroids generated from transfected LNCaP cells was investigated. Spheroids are better able to mimic the complex PCa tumor structure compared to adherent cell cultures. They reconstruct the tumor microenvironment, allow enhanced cell-cell interactions and provide a different exposure of drug molecules to the cells [37, 38]. Growth analysis of the spheroids suggests that the volume of spheroids with *MIR503HG* knockdown was significantly decreased in the control treated spheroids (Fig. 3A, B). Importantly, the spheroid size was further decreased by SAL treatment in knockdown spheroids. In line with this observation, the immunofluorescence results showed that the proliferation marker Ki67 was decreased in si*MIR503HG*-transfected spheroids treated with SAL (Fig. 3C, D). Along with the growth-inhibiting effects, staining of spheroids for SA β-Gal revealed an increased activity in knockdown spheroids treated with. SAL compared to control spheroids (Fig. 3A), confirming the results observed in monolayer cell culture.

### *MIR503HG* controls the cell-cycle and suppresses the SAL-mediated cellular senescence through the p15-pRb-E2F1 pathway

To identify the pathway by which *MIR503HG* inhibits cellular senescence, the expression levels of important cell cycle regulators were evaluated by Western blotting and qRT-PCR. The cell cycle inhibitor p15^INK4b^ inhibits the phosphorylation of pRb, which leads to downregulation of E2F1 transactivation and decreased *E2F1* mRNA [39, 40]. Previous studies reported that SAL induces cellular senescence in PCa cells by upregulation of p15^INK4b^, hypophosphorylation of pRb and downregulation of the E2F1 downstream target Cyclin D1 [7, 11].

**Fig. 4:**
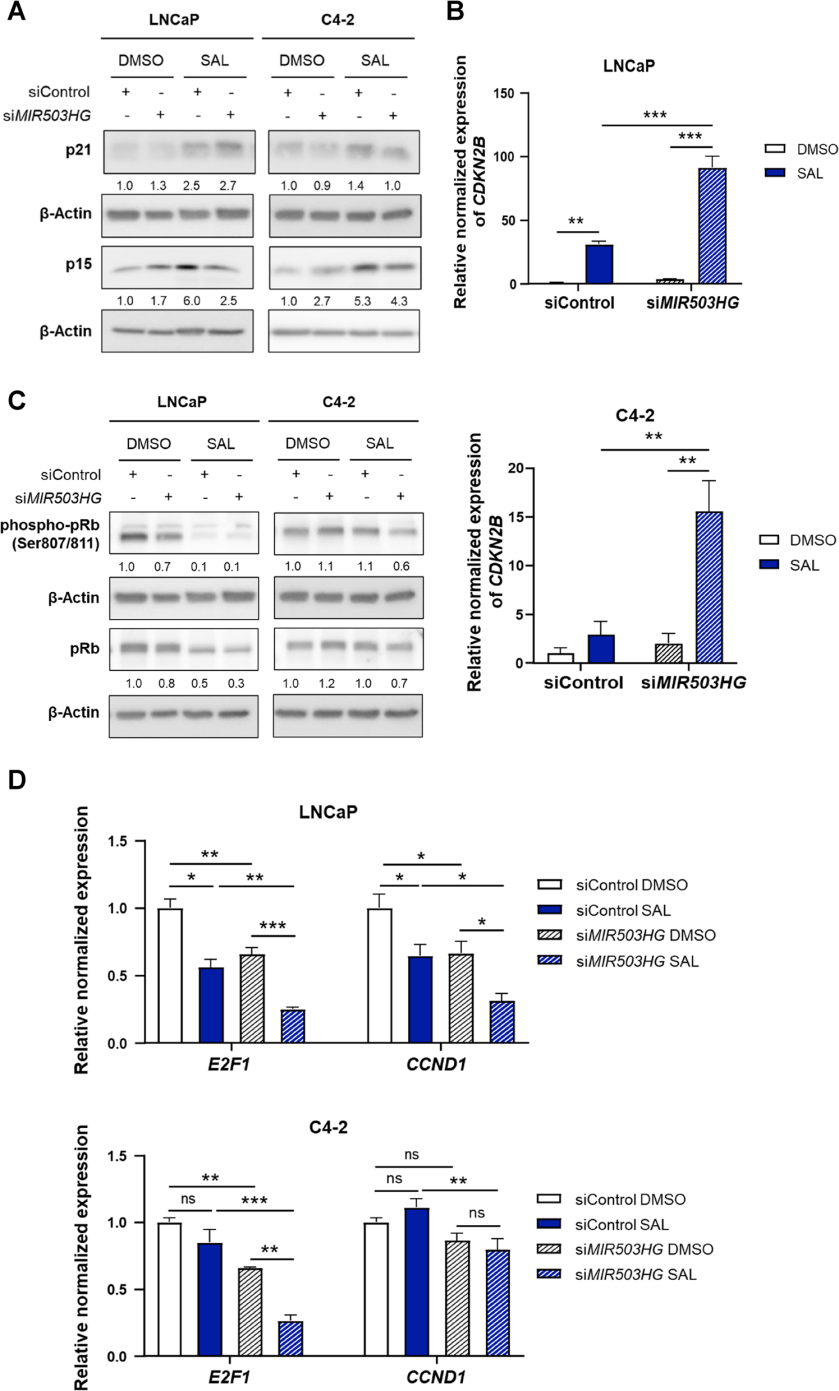
MIR503HG counteracts the SAL-mediated cellular senescence via the p15-pRb-E2F1 pathway. LNCaP and C4-2 were transfected with non-targeting siRNA (siControl) or siRNA targeting *MIR503HG* (si*MIR503HG*) and treated with DMSO or SAL. **A** Analysis of p21^WAF1/Cip1^ and p15^INK4b^ protein levels of lncRNA *MIR503HG* knockdown cells by Western blot. Numbers indicate the band intensities normalized to the loading control β-Actin (*n* = 3). **B** qRT-PCR analysis of *CDKN2B* expression normalized to *TBP* and *α-Tubulin* (*n* = 3). **C** Western blot analysis detecting changes in phospho-pRb (Ser807/811) and pan pRb levels in both cell lines. **D** Analysis of *E2F1* and *CCND1* mRNA level of transfected LNCaP and C4-2 cells normalized to *TBP* and *Tubulin* (*n* = 3). Bar graphs represent mean ± SEM (*p ≤ 0.05, **p ≤ 0.01, ***p ≤ 0.001, ns: not significant)

We hypothesized that *MIR503HG* suppresses the SAL-mediated cellular senescence through interfering with these targets. Knockdown of *MIR503HG* increased p21^WAF1/Cip1^ and p15^INK4b^ protein level in LNCaP cells, whereas only p15^INK4b^ levels were elevated in C4-2 cells (Fig. 4A). In line with this, SAL-induced *CDKN2B* (p15^INK4b^) mRNA levels were further induced by *MIR503HG* knockdown in LNCaP and C4-2 cells compared to control transfected cells (Fig. 4B). The enhanced *CDKN1B* level after 48 h treatment in the knockdown samples suggest a time-dependent effect on p15 protein level detected after 72 h, which may be due to changes in protein stability. The protein levels of p27 were not substantially changed by knockdown of *MIR503HG* (Fig. S3).

*MIR503HG* depletion also resulted in hypophosphorylation of pRb in control treated LNCaP cells (Fig. 4C), supporting the hypothesis that *MIR503HG* regulates the SAL-mediated induction of cellular senescence. Accordingly, the expression of the SAL-suppressed cell-cycle regulator *E2F1* and its downstream target *CCND1* (Cyclin D1) were further decreased in LNCaP knockdown cells (Fig. 4D).

SAL treatment of knockdown cells further enhanced the inhibitory effects on the expression level in both cell lines. Surprisingly, C4-2 cells were less sensitive to the SAL-induced hypophosphorylation of p-Rb, downregulation of *E2F1 *and *CCND1 *(Fig. 4D), which might explain the weaker effects of the knockdown on this pathway in the CRPC cells.

The results indicate that *MIR503HG* is an upstream regulator of the p15-pRb-E2F1 pathway to suppress the SAL-mediated cellular senescence.

### *MIR503HG* inhibits the AKT-p70S6K-S6 pathway to overcome SAL-mediated cellular senescence of PCa cells

**Fig. 5:**
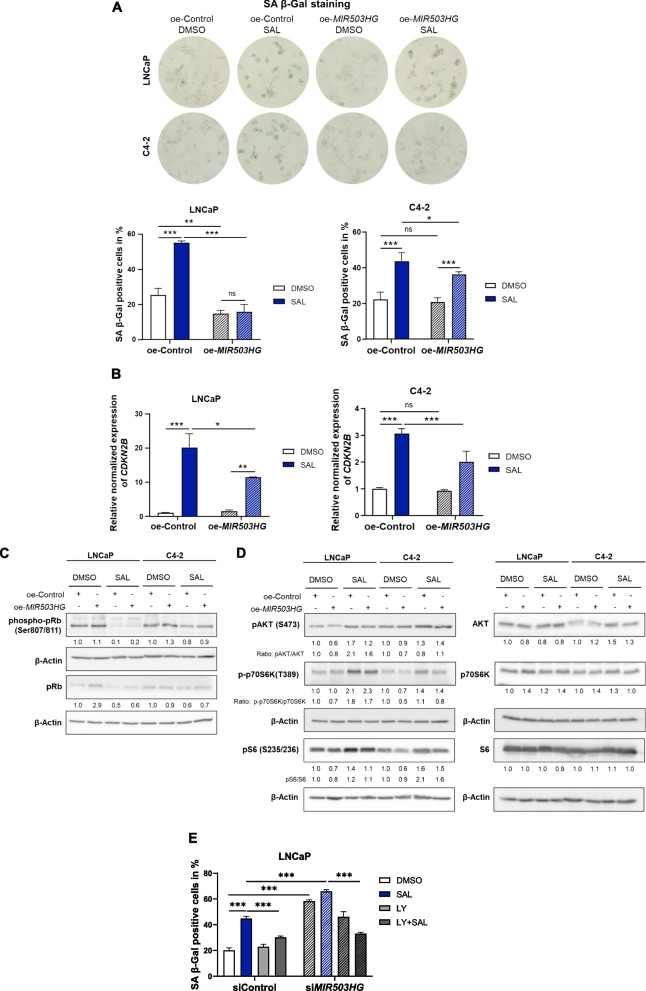
Overexpression of *MIR503HG* suppresses SAL-mediated cellular senescence of PCa cells by inhibiting the AKT-p70S6K-S6 pathway*.* LNCaP and C4-2 were transfected with empty vector control (oe-Control) or vector containing the full-length sequence of *MIR503HG* (oe-*MIR503HG*) followed by DMSO or SAL treatment for 72h. **A** SA β-Gal staining of transfected LNCaP and C4-2 cells, analyzed by phase microscopy. Two-way ANOVA was performed for multiple comparisons. Bar graphs represent mean ± SD (*n* =3). **B** Expression level of *CDKN2B *of transfected LNCaP and C4-2 cells. (*p ≤0.05, **p ≤0.01, ***p ≤0.001, ns: not significant). **C** Analysis of phospho-pRb (Ser807/811) and pRb protein levels of *MIR503HG* overexpressed cells by Western blot. **D** Protein levels ofp-AKT (S473), pan-AKT, p-p70S6K (T389), pan-p70S6K, p-S6 (Ser 235/236) and pan-S6 were assessed by Western blotting. Numbers indicate the band intensities normalized to the loading control β-Actin (*n* =3). **E** Senescence level of transfected LNCaP treated with SAL alone or in combination with the p70S6K inhibitor LY2584702 (3 µM)

To further confirm the inhibitory activity of lncRNA *MIR503HG* on PCa cell senescence, we transfected LNCaP and C4-2 cells with a pcDNA3.1 vector containing full-length *MIR503HG* (oe-*MIR503HG*) or a non-targeting control vector (oe-Control). The results showed that overexpression of *MIR503HG* decreased cellular senescence level, particularly in SAL-treated cells (Fig. 5A), confirming that *MIR503HG* inhibits cellular senescence in cancer. The observation that *MIR503HG* downregulates SAL-induced *CDKN2B* expression level in both cell lines (Fig. 5B) further supports the findings that *MIR503HG* is part of androgen signaling. In contrast to knockdown experiments, *MIR503HG* overexpression moderately increased the phospho-pRb and total pRb level in control and SAL treated LNCaP and C4-2 cells (Fig. 5C). The overexpression experiments confirm the suppressive effects of *MIR503HG* on cellular senescence.

The PI3K/AKT signaling pathway is a major pro-survival pathway in cancers [41, 42]. Notably, the AKT pathway plays a pivotal role in mediating the SAL-induced cellular senescence [11]. We observed a negative correlation between *MIR503HG* expression and *AKT1* expression (Supplemental Table S5). In line with this, overexpression of *MIR503HG* decreased phosphorylation level of AKT (S473) in LNCaP and to less extend in C4-2 cells (Fig. 5D). Phosphorylation levels of the AKT downstream targets p70S6K and S6 were also reduced in *MIR503HG* overexpressed cells, particularly in SAL treated overexpressed cells. To confirm that *MIR503HG* regulates cellular senescence through the AKT-p70S6K-S6 pathway, *MIR503HG* knockdown cells were treated with SAL alone or in combination with the p70S6K inhibitor LY2584702. Inhibition of the p70S6K partially rescued the induction of cellular senescence by the knockdown of the lncRNA in the presence of SAL (Fig. 5E). The results propose an additional pathway through which *MIR503HG* regulates cellular senescence and survival of PCa cells.

### *MIR503HG* controls the expression of SAL-regulated AR target genes

**Fig. 6:**
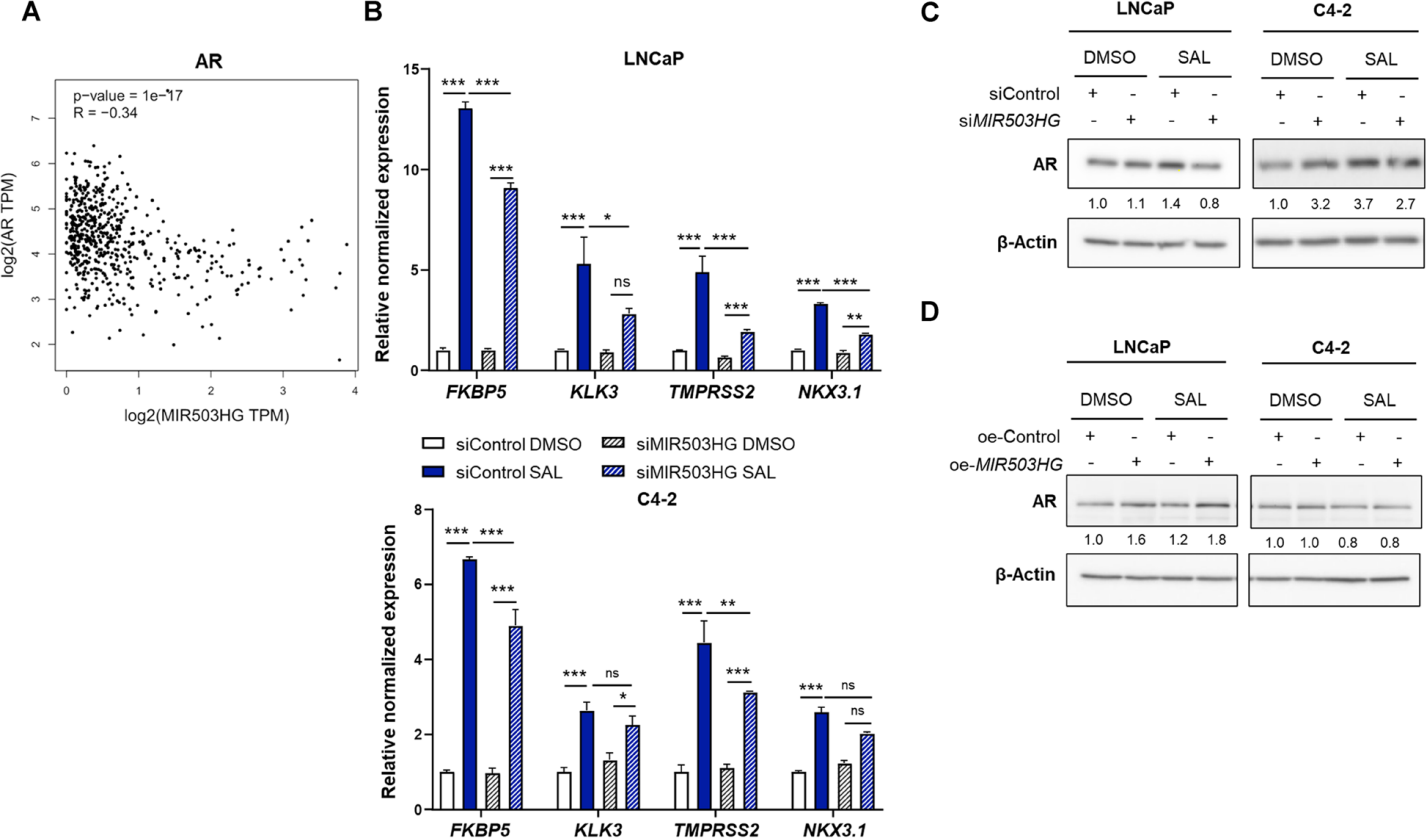
*MIR503HG* enhances the expression of SAL-regulated AR target genes. **A** Correlation of *MIR503HG* and *AR *expression using TCGA PRAD and GTEx prostate datasets from GEPIA. Spearman correlation coefficient was calculated (*R* = -0.34, *p* - value = 1e^-17^). **B** Expression levels of the AR target genes *FKBP5*, *KLK3, TMPRSS2 and NKX3.1 *following knockdown of *MIR503HG* with siRNA and treatment with SAL or DMSO for 48h. Bar graphs represent mean ± SEM (*n* =3) (*p ≤0.05, **p ≤0.01, ***p ≤0.001, ns: not significant). **C** AR protein levels of *MIR503HG* knockdown and overexpressing cells (**D**) were analyzed by Western blotting. Numbers indicate the band intensities normalized to the loading control β-Actin (*n* =3)

The correlation analysis of *MIR503HG* expression with different nuclear hormone receptors in prostate cancer specimen obtained from TCGA PRAD and GTEx prostate datasets revealed that *MIR503HG* significantly and negatively correlates with *AR* expression (Fig. 6A), while only a weak and rather positive correlation with other steroid hormone receptors including *ESR1*,* PRH2*, *NR3C1*, *NR3C2*,* VDR* and *THRA* exists (Fig. S4A) suggesting a specific inverse correlation between *MIR503HG* and *AR.* This specificity supports the observed repression of *MIR503HG* by AR and implies a targeted role for *MIR503HG* in modulating specifically AR-related pathways in PCa. To further address whether *MIR503HG* is involved in AR signaling, the expression of the positively regulated AR target genes *FKBP5*,* KLK3* (PSA), *TMPRSS2* and *NKX3.1* was analyzed. Interestingly, the SAL-induced expression of *FKBP5*, *KLK3*,* TMPRSS2* and *NKX3.1* was significantly repressed by knockdown of *MIR503HG* in LNCaP cells (Fig. 6B). These findings demonstrate that *MIR503HG* is involved in SAL-regulated transcriptional activity of AR. Next, AR protein levels were examined after knockdown and overexpression of *MIR503HG* by Western blot. In both cell lines, SAL-enhanced AR protein levels were decreased in knockdown cells, being more pronounced in LNCaP cells (Fig. 6C), accounting for the decreased transcriptional activity of AR by knockdown of *MIR503HG*.

Overexpression of *MIR503HG* resulted in increased expression of the AR target gene *FKBP5* upon SAL treatment (Fig. S4C) and elevated AR protein levels in SAL treated LNCaP cells (Fig. 6D) compared to control-transfected cells. This further supports a crosstalk between *MIR503HG* and the AR treating cells with SAL. In line with this, we observed in PDX specimen a positive correlation between *MIR503HG* and *FKBP5* RNA counts in publicly available RNA-Seq data of PDX treated with supraphysiological testosterone (Fig. S4B), confirming our expression data. These results suggest that *MIR503HG* is involved in the AR signaling and imply a novel negative feedback loop in the AR signaling.

### *MIR503HG* inhibits miR-424-5p to suppress expression of *CDKN2B* mitigating cellular senescence

**Fig. 7:**
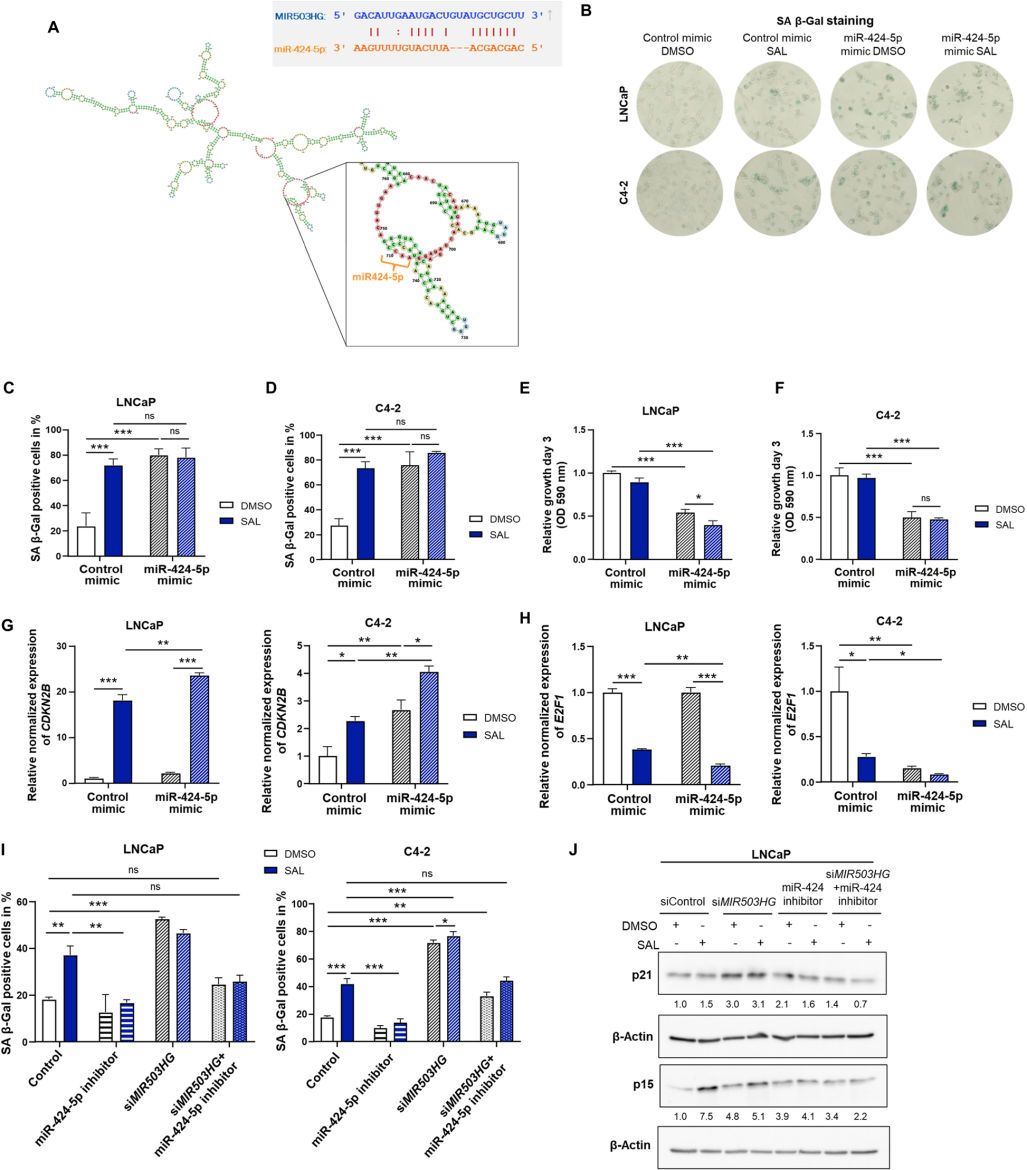
*MIR503HG* sponges miR-424-5p to suppress expression of *CDKN2B* and to inhibit senescence. **A** Predicted binding site of *MIR503HG* with miR-424-5p. Binding of miR-424-5p to the predicted secondary structure of lncRNA *MIR503HG* is represented with enlarged view of the binding loop. **B**-**H** LNCaP and C4-2 were transfected with miR-424-5p mimic or microRNA mimic negative control (control) and treated with DMSO or SAL for 48 h. **B** SA β-Gal staining of transfected LNCaP and C4-2 cells, analyzed by phase microscopy. **C**, **D** Quantification of SA β-Gal positive cells in both cell lines. Two-way ANOVA was performed for multiple comparisons (*n* = 3) (***p ≤0.001, ns: not significant). **E**, **F** Growth of miR-424-5p or control transfected LNCaP and C4-2 cells (*n* =3). **G**, **H** Transfected cells were analyzed for mRNA levels of *CDKN2B* and *E2F1* (*n* = 3) (*p ≤0.05, **p ≤0.01, ***p ≤0.001, ns: not significant). **I** SA β-Gal activity was examined after transfection of LNCaP and C4-2 cells with si*MIR503HG* and/or miR-424-5p inhibitor and treatment for 48h. Two-way ANOVA was performed for multiple comparisons (*n* = 3). **J** Detection of p15 and p21 protein levels of si*MIR503HG* and/or miR-424-5p inhibitor transfected LNCaP cells after 72 h treatment with DMSO or SAL. Numbers indicate the band intensities normalized to the loading control β-Actin (*n* = 3)

The miR-424-5p was predicted as target of *MIR503HG* by the starBase database with a low minimum free energy of −22.5 kcal/mol for binding (Fig. 7A). Previous studies in endothelial cells showed that *MIR503HG* knockdown upregulates the expression of *miR-424* [43]. In normal human mammary epithelial cells, the miR-424 has been described as a regulator of the p16-mediated cellular senescence by inhibiting the expression of the polycomb group (PcG) proteins CBX7, embryonic ectoderm development (EED), enhancer of zeste homologue 2 (EZH2) and suppressor of zeste 12 homologue (Suz12) [44].

Therefore, we hypothesized that *MIR503HG* regulates androgen-induced cellular senescence and growth of PCa cells through miR-424. To analyze the effects of miR-424 on cellular senescence, PCa cells were transfected with miR-424-5p mimic or mimic negative control (control mimic), respectively. The results indicate that upregulation of miR-424-5p effectively induced cellular senescence independent of SAL treatment in both cell lines (Fig. 7B, C, D). Notably, cellular senescence levels were not elevated by the miR-424-3p mimic as a further control, indicating a specific role of miR-424-5p in PCa cell senescence (Fig. S5A). Moreover, the growth of both cell lines was potently reduced in miR-424-5p transfected cells by control as well as by SAL treatment (Fig. 7E, F, Fig. S5B). To confirm whether miR-424-5p act through the same cellular pathway to regulate cellular senescence, changes in mRNA level of *CDKN2B* and *E2F1* were analyzed (Fig. 7G, H). miR-424-5p mimic increased the SAL-induced expression of *CDKN2B* and decreased the SAL-repressed expression of *E2F1*, however effects were less pronounced compared to *MIR503HG* knockdown.

We further tested whether miR-424-5p represses the expression of the PcG member *EZH2* therefore leading to *CDKN2B* expression. Indeed, miR-424-5p mimic repressed the expression of *EZH2* in control and SAL treated LNCaP and C4-2 cells (Fig. S5C). In conclusion, these results suggest that miR-424-5p drives cellular senescence by repressing EZH2 and modulation of *CDKN2B* expression.

To investigate whether inhibition of miR-424-5p is sufficient to prevent induction of cellular senescence by *MIR503HG* knockdown, miR-424-5p inhibitor or control inhibitor were co-transfected with si*MIR503HG* respectively to LNCaP and C4-2 cells in a rescue experiment. The results demonstrate that the SAL-mediated cellular senescence level was significantly decreased by the miR-424-5p inhibitor. The data indicate that the miR-424-5p inhibitor reversed the induction of cellular senescence mediated by knockdown of *MIR503HG* in both PCa cell lines indicating that miR-424 plays a critical role in mediating senescence by the *MIR503HG* suppression (Fig. 7I). The experiment demonstrates that miR-424-5p is downstream of the *MIR503HG* regulatory pathway controlling cellular senescence. Furthermore, the presence of miR-424-5p inhibitor reversed the effects of *MIR503HG* knockdown on p15 and p21 protein levels (Fig. 7J). According to the results, it suggests that *MIR503HG* inhibits cellular senescence of PCa cells by targeting and inhibiting miR-424-5p.

### *MIR503HG *upregulates the expression of the SAL-repressed DNA repair gene *BRCA2*

Recent studies have shown that SAL might exert its clinical effects by inducing double-strand DNA breaks and repressing genes involved in DNA repair processes, particularly in breast cancer type 2 susceptibility protein (BRCA2)-deficient PCa patients [45]. Further, poly (ADP-ribose) polymerase (PARP)-mediated repair pathways are upregulated in PCa following androgen-deprivation therapy [46]. In line with this, using the combination of SAL with PARP inhibitor further enhanced the effects on DNA damage and growth suppression [45]. These findings provide a rational for combining BAT with the PARP inhibitor Olaparib, which is currently tested in an ongoing clinal trial for patients with metastatic CRPC [29].

**Fig. 8:**
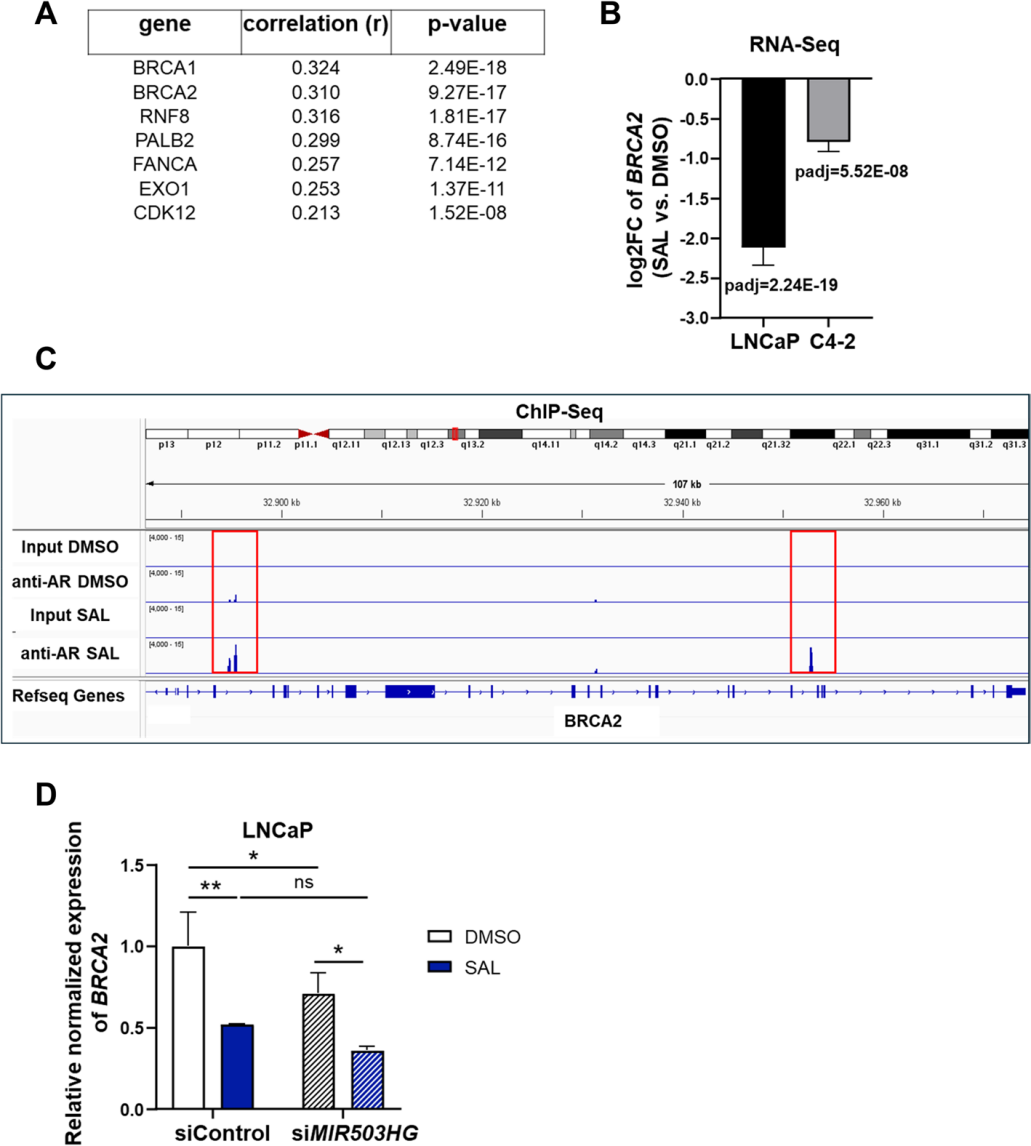
*MIR503HG* regulates SAL-repressed *BRCA2* expression. **A** Positive correlation between *MIR503HG* and the DNA repair genes *BRCA1, BRCA2, PALB2, FANCA, CDK12 *in TCGA RNA-Seq samples of normal tissue, primary and metastatic PCa tumors. **B** RNA-Seq data were analyzed for expression changes of *BRCA2* by SAL treatment in LNCaP and C4-2 cells compared to control treatment. **C** ChIP-Seq of AR was conducted in LNCaP cells and the SAL-induced recruitment of AR to the *BRCA2* locus was detected and visualized by IGV software. Input served as negative control (*n* = 3). **D** Assessment of*BRCA2 *mRNA expression after knockdown of *MIR503HG *in LNCaP cells. Bar graphs represent mean ± SEM (*n* = 3) (*p ≤0.05, **p ≤0.01, ns: not significant)

Interestingly, our correlation analysis revealed a positive correlation between the expression of *MIR503HG* and the DNA repair genes *BRCA1*,* BRCA2*,* RNF8*,* PALB2*,* FANCA*,* EXO1*,* CDK12* (Fig. 8A). Analyzing our RNA-Seq data suggests that the expression of the DNA repair gene *BRCA2* was significantly suppressed by SAL treatment (Fig. 8B) in both androgen-dependent and independent PCa cell lines, confirming aforementioned previous studies. Notably, the AR is directly recruited to the *BRCA2*,* RNF8* and *EXO1* locus in a SAL-dependent manner (Fig. 8C, Fig. S6), concluding that these genes are novel direct AR repressed target genes.

Based on the obtained correlation data, we hypothesized that *MIR503HG* influences the *BRCA2* gene expression, thereby modulating DNA repair mechanisms and mediating the tumor-promoting effects. Therefore, we addressed the question whether *MIR503HG* regulates *BRCA2* expression. Since SAL downregulates *MIR503HG*, we hypothesized that in the absence of SAL, the knockdown of *MIR503HG* reduces *BRCA2* mRNA levels. Indeed, knockdown experiments of *MIR503HG* resulted in downregulation of *BRCA2* mRNA expression, an effect further augmented by SAL treatment (Fig. 8D). These results suggest that *MIR503HG* promotes the expression of *BRCA2*, potentially providing a survival advantage for PCa cells. Thus, the findings provide an underlaying molecular basis of combination therapy with SAL and PARP inhibition. In conclusion, our findings highlight a novel pathway in AR signaling and an interplay between *MIR503HG*, BRCA2, and SAL in modulating DNA repair mechanisms and induction of cellular senescence in PCa.

## Discussion

Recent advances in PCa treatment have highlighted the potential of SAL to induce cellular senescence as a tumor suppressive mechanism, which is exploited in the bipolar androgen therapy [7, 11, 12]. Accumulating findings suggest that lncRNAs play pivotal roles in regulating cellular senescence pathways [22, 47]. For example, the lncRNA *ANRIL* (Antisense noncoding RNA in the INK4 locus) is downregulated in replicative and Ras-induced senescence, which relieves the transcriptional repression of the *CDKN2A/CDKN2B* locus by the polycomb repressive complexes 1 and 2 [48–50]. Further, cellular senescence mediated by p21^WAF1/Cip1^ is suppressed by the lncRNA *GUARDIN* through a LRP130-PGC1a-FOXO4 signaling axis in various cancer types [51] whereas p21^WAF1/Cip1^ plays rather an ancillary role in inducing SAL-mediated senescence in PCa. However, the role of lncRNAs in the regulation of the SAL-mediated cellular senescence and the molecular mechanisms underlying the BAT remains largely unknown.

Our transcriptome analysis indicates that the SAL-induced cellular senescence is associated with changes in the expression of lncRNAs, with a substantial number being commonly downregulated by SAL treatment in CSPC and CRPC cell lines, suggesting that AR may exert a direct or indirect transcriptional repression of these lncRNAs. Mechanistically, AR is known to regulate target genes both positively and negatively. It has been shown that AR represses the transcription of the human telomerase reverse transcriptase (hTERT) in response to SAL by binding to the TERT promoter [52]. TERT, a key component of telomerase, has been shown to prevent cell senescence and facilitates uncontrolled proliferation [53]. Thus, the transcriptional repressor activity of AR might provide one tumor suppressive mechanism by inhibiting potential pro-tumorigenic genes [54, 55]. Therefore, repression of lncRNAs by AR might be critical in mediating the cellular responses to SAL treatment, contributing to the overall senescence phenotype observed.

In this study, we identified the lncRNA *MIR503HG* as a novel SAL-repressed gene with potential oncogenic activity in PCa that inhibits androgen-mediated cellular senescence. The repression of *MIR503HG* by SAL is consistent across in vitro cell lines, in vivo CRPC xenografts, and PDX models.

Interestingly, *MIR503HG* shows aberrant expression in different cancer types [33]. *MIR503HG* is downregulated in hepatocellular carcinoma, gastric cancer, colon cancer, and ovarian cancer indicating a potential tumor suppressive role in these cancer types [25, 27, 28]. Conversely, in anaplastic lymphoma kinase-negative anaplastic large cell-lymphoma (ALK-negative ALCL) and non-small cell lung cancer *MIR503HG* is upregulated and mediates tumor-promoting effects [56, 57]. This implies, that *MIR503HG* has cancer type-specific effects and can function as tumor suppressor or oncogene.

Our data revealed that *MIR503HG* is significantly overexpressed in metastatic PCa tumors compared to normal tissue, which correlates with significantly reduced overall and disease-free survival of patients with prostate adenocarcinoma. These findings suggest that *MIR503HG* acts as an oncogenic regulator in PCa. Accordingly, low *MIR503HG* expression is associated with SAL-responsiveness in xenograft tumors. Therefore, *MIR503HG* may represent a prognostic biomarker, a biomarker for the treatment response to BAT and for predicting patient survival in PCa.

The GO enrichment analysis of TCGA datasets further demonstrates that *MIR503HG* expression positively correlates with genes involved in pro-tumorigenic pathways suggesting that *MIR503HG* promotes cancer cell survival. In line with it, functional assays showed that knockdown of *MIR503HG* potently suppressed the growth of both CRPC and CSPC cell lines and tumor spheroids indicating that *MIR503HG* promotes PCa growth. These findings are consistent with previous studies showing that *MIR503HG* enhances tumor cell proliferation of ALK-negative ALCL [56].

Intriguingly, we have shown that depletion of *MIR503HG* is pivotal for the induction of cellular senescence by SAL, while overexpression inhibited cellular senescence of CSPC and CRPC cells. Hence, our data highlight *MIR503HG* as a potent suppressor of the SAL-induced cellular senescence. Investigation of the underlying mechanisms revealed that *MIR503HG* knockdown resulted in the upregulation of the cell cycle inhibitors p15^INK4b^ and p21^WAF1/Cip1^, hypophosphorylation of pRb, and downregulation of *E2F1* and *Cyclin D1*, leading to cell cycle arrest and senescence. Consistently, the p15-pRb-E2F1 pathway was previously shown to mediate cellular senescence in response to SAL [11].

According to the findings that the AKT pathway plays a critical role in mediating SAL-induced senescence [11], overexpression of *MIR503HG* inhibits AKT phosphorylation and activation by SAL, thereby attenuating cellular senescence. The importance of *MIR503HG* in the negative regulation of the AKT pathway was also reported in ovarian cancer [28]. The partial reversal of cellular senescence upon inhibition of the p70S6K using the inhibitor LY2584702 in *MIR503HG* knockdown cells treated with SAL supports the notion that the lncRNA influences cellular senescence via the AKT-p70S6K-S6 pathway.

The aberrant regulation of lncRNA-miRNA networks play an important role in tumorigenesis and cancer progression [58, 59]. In these networks, lncRNAs can act as competing endogenous RNAs (ceRNAs) sponges, interacting with miRNAs to remove their inhibitory effect on specific downstream target genes [60, 61]. In general, miRNAs regulate the gene expression by targeting their mRNAs resulting in the degradation or translational inhibition [62]. We identified miR-424-5p as a downstream target of *MIR503HG*, involved in regulating cellular senescence. Transfection of the miR-424-5p mimic, but not the miR-424-3p mimic, induced senescence and suppressed PCa cell growth, supporting the notion that *MIR503HG* inhibits miR-424-5p to prevent senescence.

Genomic alterations in DNA damage response (DDR) genes are commonly found in patients with metastatic CRPC [63, 64]. These alterations are of therapeutic interest because they determine the responsiveness of tumors to PARP inhibitors [65–67].

Interestingly, our bioinformatic correlation analysis provides evidence that *MIR503HG*, in addition to its role in promoting cancer cell proliferation, also regulates various DNA repair factors. Our data demonstrate a positive correlation between *MIR503HG* and the expression of genes mediating homology-directed DNA repair, such as *BRCA2*. Importantly, knockdown of *MIR503HG* resulted in decreased *BRCA2* mRNA levels, suggesting that *MIR503HG* promotes *BRCA2* expression. This upregulation of *BRCA2* potentially provides a survival advantage to PCa cells by enhancing their ability to repair DNA damage, thereby contributing to tumorigenesis and resistance to therapy. Noteworthy, patients with BRCA alterations have significantly worse progression-free survival [64].

The suppression of *MIR503HG* by SAL not only induces cellular senescence but also downregulates *BRCA2*, impairing the DNA repair capacity of PCa cells. Thus, the interplay between *MIR503HG*,* BRCA2*, and androgen signaling underscores a novel pathway in AR signaling. This dual action of SAL on cellular senescence and DNA repair highlights the potential benefit of combining SAL with PARP inhibitors, which target BRCA2-deficient cells, for enhanced therapeutic efficacy. Our data point towards the notion that the *MIR503HG* has the potential as a diagnostic biomarker for the responsiveness to BAT therapy, particularly in combination with PARP inhibitors. By exploiting the vulnerabilities introduced by *MIR503HG* suppression, such combination therapies could provide a powerful strategy to overcome resistance and improve outcomes for patients with advanced PCa. A phase II clinical trial combines BAT with Olaparib, a PARP inhibitor, in men with metastatic CRPC [29]. Of note, this combination therapy is associated with high response rates and long progression-free survival. Importantly and in accordance with our suggested molecular mechanism, there is a clinical benefit regardless of homologous recombination repair gene mutations, which includes BRCA2.

## Conclusions

**Fig. 9:**
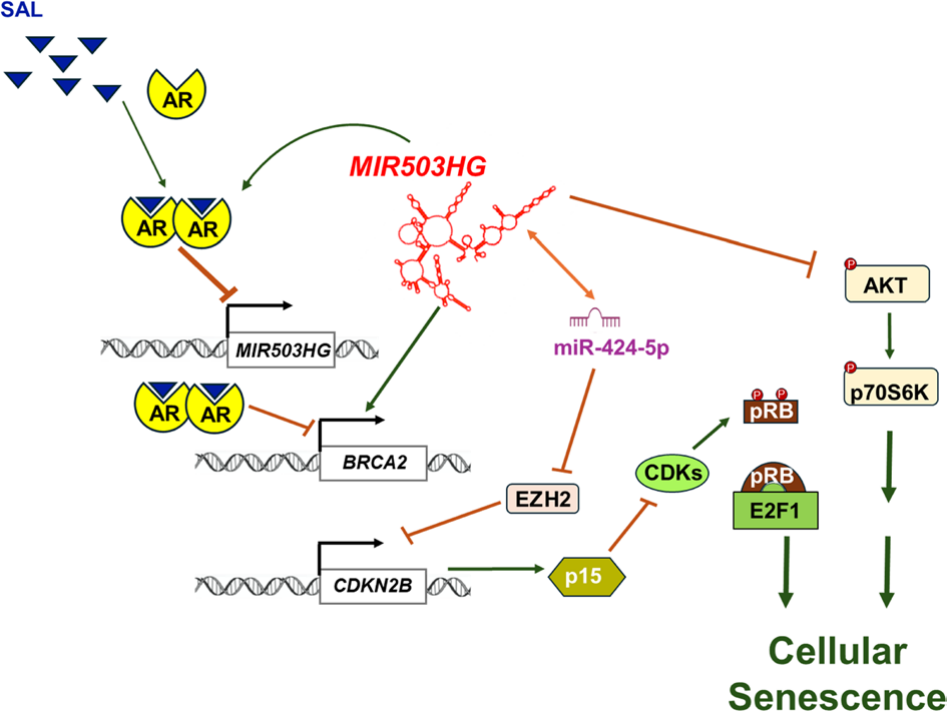
*MIR503HG* suppresses SAL-mediated cellular senescence through miR-424-5p and upregulates *BRCA2*.SAL represses lncRNA *MIR503HG *expression relieving the inhibition of miR-424-5p. miR-424-5p inhibits the polycomb group protein EZH2, thereby activating p15 and leading to hypophosphorylation of pRb, downregulation of E2F1 transactivation and induction of cellular senescence.  In addition, *MIR503HG* inhibits cellular senescence through the AKT-p70S6K pathway. The expression of the SAL-repressed DNA damage response gene *BRCA2* is induced by the lncRNA. The figure was partially created using BioRender.com with modifications

In conclusion, our data suggest that the lncRNA *MIR503HG* has oncogenic activity in PCa by interfering with SAL-induced cellular senescence through miR-424-5p and upregulation of *BRCA2* (Fig. 9). These findings reveal a novel senescence-regulating AR-*MIR503HG*-miR-424-5p signaling axis and a link between SAL-*MIR503HG* and homology-directed DNA repair through BRCA2. Furthermore, *MIR503HG* modulates key pathways such as p15^INK4b^-pRb-E2F1 and AKT-p70S6K-S6. These findings highlight the potential of targeting *MIR503HG* as a therapeutic strategy in PCa, particularly in enhancing the efficacy of SAL-induced cellular senescence and DNA repair inhibition in BAT and in combination treatment with PARP inhibitors.

## Supplementary Information

Supplementary Material 1.

Supplementary Material 2.

Supplementary Material 3.

Supplementary Material 4.

Supplementary Material 5.

## Data Availability

The RNA-Seq datasets from LNCaP, C4-2, VCaP, LAPC4 cells, and PDX xenografts analyzed in this study are accessible in GEO under accession numbers GSE155528, GSE172205, GSE225481, and GSE188174, respectively. The TCGA PRAD and GTEx prostate datasets from GEPIA were used for correlation analysis and survival plots of prostate adenocarcinoma patients (assessed on 04 July 2024). The starBase database was used for prediction of miR-424-5p binding site in *MIR503HG*.

## Abbreviations

AR: androgen receptor
BAT: bipolar androgen therapy
BRCA2: breast cancer type 2 susceptibility protein
ceRNA: competing endogenous RNAs
ChIP-Seq: chromatin immunoprecipitation sequencing
CRPC: castration resistant prostate cancer
CSPC: castration sensitive prostate cancer
DDR: DNA damage response
GEPIA: Gene Expression Profiling Interactive Analysis
GO: gene ontology
lncRNA: long non-coding RNA
PARP: poly (ADP-ribose) polymerase
PCa: prostate cancer
PcG: polycomb group
PDX: patient-derived xenograft
PRAD: prostate adenocarcinoma
SA β-Gal: senescence-associated beta-galactosidase
SAL: supraphysiological androgen level
TCGA: The Cancer Genome Atlas
TERT: telomerase reverse transcriptase
