# Supplementary material for: The oncogenic lncRNA MIR503HG suppresses cellular senescence counteracting supraphysiological androgen treatment in prostate cancer
Source: J Exp Clin Cancer Res. 2024 Dec 16;43:321. doi: 10.1186/s13046-024-03233-2 (PMC11648305; doi:10.1186/s13046-024-03233-2)
Supplement: Supplementary file 2 — Supplementary Material 2. [file 13046_2024_3233_MOESM2_ESM.docx]

**Supplementary tables**

| **Supplement Table 1. Sequences of siRNAs** | |
| --- | --- |
| **Target** | **Sequence** |
| Lincode Non-targeting Pool (siControl) | UGGUUUACAUGUCGACUAA, UGGUUUACAUGUUGUGUGA, UGGUUUACAUGUUUUCUGA, UGGUUUACAUGUUUUCCUA |
| Lincode Human MIR503HG (84848) SMARTpool siRNA | GUCCCAAAUAGAAGGGUAA, CCGCCAAAUGAGUCAGUCA, UGUUCCAAUUGGCAUGUAA, AAGGAACACUACAUCAACA |

| **Supplement Table 2. Sequence of primers used in qRT-PCR (5’-3’)** | |
| --- | --- |
| **Target** | **Sequence (5’-3’)** |
| *TBP* | FW: GATCTTTGCAGTGACCCAGCATCA REV: CTCCAGCACACTCTTCTCAGC |
| *α-Tubulin* | FW: TGGAACCCACAGTCATTGATGA REV: TGATCTCCTTGCCAATGGTGTA |
| *MIR503HG* | FW: TTCCTGAAAGACCAAGCCCG REV: TGGAGATGCTGGATGCCTTC |
| *CDKN2B* | FW: GAATGCGCGAGGAGAACAAG REV: TCATCATGACCTGGATCGCG |
| *E2F1* | FW: GCAGAGCAGATGGTTATGG REV: GATCTGAAAGTTCTCCGAAGAG |
| *CCND1* | FW: TGAACTACCTGGACCGCTTC REV: CCACTTGAGCTTGTTCACCA |
| *FKBP5* | FW: GAGGAAACGCCGATGATTGGAGAC REV: CATGCCTTGATGACTTGGCCTTTG |
| *KLK3* | FW: GAGGCTGGGAGTGCGAGAAG REV: TTGTTCCTGATGCAGTGGGC |

| Supplement Table 3. Antibodies | | | | |
| --- | --- | --- | --- | --- |
| Target antigen | **dilution** | **application** | **company** | **Cat No.** |
| anti-AR | 1:1000 | Western blotting | Merck Millipore | #06-680 |
| anti-phospho-Rb (Ser807/811) | 1:500 | Western blotting | Cell Signaling | #9308 |
| anti-Rb | 1:500 | Western blotting | Abcam | ab6075 |
| anti-p21WAF1/Cip1 | 1:1000 | Western blotting | Cell Signaling | #2946 |
| anti-p15INK4b | 1:2000 | Western blotting | MyBioSource | MBS821044 |
| anti-P70S6K | 1:1000 | Western blotting | Cell Signaling | #2708 |
| anti-p-P70S6K(T389) | 1:1000 | Western blotting | Cell Signaling | #9205 |
| anti-β-Actin | 1:10,000 | Western blotting | Abcam | ab6276 |
| anti-Pan-AKT | 1:5000 | Western blotting | Cell Signaling | #4685 |
| anti-p-AKT(S473) | 1:5000 | Western blotting | Cell Signaling | #4058 |
| anti-mouse IgG | 1:10,000 | Western blotting | Cell Signaling | #7076S |
| anti-rabbit IgG | 1:10,000 | Western blotting | Cell Signaling | #7074S |
| anti-Ki67 | 1:200 | Immunofluorescence | Biozol | VP-RM04 |
| anti-rabbit IgG Alexa 546 | 1:1000 | Immunofluorescence | Invitrogen | A11035 |
| anti-AR | 1µg | ChIP-Seq | Cell Signaling | #5153 |

Supplement Table 4. GO pathways enriched for genes positively correlated with *MIR503HG* expression. Correlation analysis was conducted with 52 normal tissue samples, 539 primary and 100 metastatic PCa tumor samples from TCGA database (genes with correlation coefficient of > 0.5 were used).

| **Pathways** | **p-value** |
| --- | --- |
| calcium-dependent cell-cell adhesion via plasma membrane cell adhesion molecules | 0.000085 |
| angiogenesis | 0.00017 |
| regulation of cell shape | 0.00018 |
| regulation of establishment of cell polarity | 0.00032 |
| **positive regulation of angiogenesis** | **0.00043** |
| regulation of cell migration | 0.00089 |
| positive regulation of mitotic cell cycle spindle assembly checkpoint | 0.00185 |
| positive regulation of vasculogenesis | 0.00185 |
| regulation of endothelial cell proliferation | 0.00231 |
| homophilic cell adhesion via plasma membrane adhesion molecules | 0.00231 |
| cardiac septum development | 0.00272 |
| **endothelial cell migration** | **0.00295** |
| lipid phosphorylation | 0.00331 |
| ganglioside biosynthetic process | 0.00374 |
| tissue homeostasis | 0.00381 |
| gap junction assembly | 0.00419 |
| regulation of focal adhesion assembly | 0.00504 |
| vascular wound healing | 0.00517 |
| regulation of positive chemotaxis | 0.00531 |
| peptidyl-tyrosine autophosphorylation | 0.00569 |
| sialylation | 0.00569 |
| nucleotide transmembrane transport | 0.00569 |
| plasma membrane bounded cell projection organization | 0.00621 |
| neuron development | 0.00667 |
| negative regulation of Rho protein signal transduction | 0.00681 |
| negative regulation of TORC1 signaling | 0.00681 |
| nitric oxide biosynthetic process | 0.00783 |
| negative regulation of response to external stimulus | 0.00799 |
| positive regulation of positive chemotaxis | 0.00802 |
| purine-containing compound transmembrane transport | 0.00802 |
| diacylglycerol metabolic process | 0.00866 |
| glomerular epithelium development | 0.00866 |
| **positive regulation of phosphatidylinositol 3-kinase signaling** | **0.00903** |
| protein kinase C-activating G protein-coupled receptor signaling pathway | 0.00932 |
| positive regulation of molecular function | 0.00994 |
| glomerulus vasculature development | 0.01 |
| **positive regulation of cell migration** | **0.01004** |
| cell-matrix adhesion | 0.01248 |
| regulation of embryonic development | 0.01285 |
| **positive regulation of cell motility** | **0.01286** |
| phosphatidic acid biosynthetic process | 0.01372 |
| maintenance of blood-brain barrier | 0.01536 |
| **negative regulation of endothelial cell apoptotic process** | **0.01536** |
| branching involved in blood vessel morphogenesis | 0.0162 |
| basement membrane organization | 0.0162 |
| axon development | 0.01688 |
| phosphatidic acid metabolic process | 0.01707 |
| positive regulation of phospholipase C activity | 0.01886 |
| regeneration | 0.01976 |
| positive regulation of BMP signaling pathway | 0.01978 |
| regulation of phospholipase C activity | 0.02072 |
| regulation of actin cytoskeleton reorganization | 0.02072 |
| positive regulation of endothelial cell proliferation | 0.02135 |
| cell-substrate adhesion | 0.02145 |
| **negative regulation of cell adhesion** | **0.02264** |
| semaphorin-plexin signaling pathway | 0.02366 |
| regulation of catalytic activity | 0.02512 |
| exocrine system development | 0.02571 |
| epithelial cell differentiation involved in kidney development | 0.02782 |
| coronary vasculature development | 0.02782 |
| vascular endothelial growth factor signaling pathway | 0.02891 |
| negative regulation of I-kappaB kinase/NF-kappaB signaling | 0.02891 |
| negative regulation of neuron death | 0.02982 |
| positive regulation of cell-substrate adhesion | 0.03039 |
| **positive regulation of epithelial cell proliferation** | **0.0307** |
| adherens junction organization | 0.03112 |
| cell morphogenesis | 0.0325 |
| cell morphogenesis involved in neuron differentiation | 0.03281 |
| cell-cell junction assembly | 0.03321 |
| blood coagulation | 0.03343 |
| inflammatory response | 0.03368 |
| positive regulation of phospholipase activity | 0.03457 |
| axon regeneration | 0.03457 |
| epithelial tube morphogenesis | 0.03504 |
| coagulation | 0.03533 |
| neuron projection development | 0.03586 |
| hemostasis | 0.0363 |
| positive regulation of cell-matrix adhesion | 0.03694 |
| vascular endothelial growth factor receptor signaling pathway | 0.03694 |
| regulation of cell adhesion | 0.03729 |
| endothelium development | 0.0376 |
| regulation of G protein-coupled receptor signaling pathway | 0.03829 |
| axon guidance | 0.0393 |
| cell differentiation involved in kidney development | 0.03938 |
| regulation of endothelial cell apoptotic process | 0.03938 |
| neuron projection guidance | 0.03982 |
| cell junction organization | 0.04065 |
| protein-containing complex disassembly | 0.04086 |
| negative regulation of protein-containing complex assembly | 0.04114 |
| digestive system development | 0.04187 |
| aorta development | 0.04188 |
| positive regulation of oxidoreductase activity | 0.04188 |
| cell morphogenesis involved in differentiation | 0.04191 |
| glycosylation | 0.04192 |
| anatomical structure morphogenesis | 0.04276 |
| neuron projection regeneration | 0.04315 |
| regulation of nitric oxide biosynthetic process | 0.04315 |
| negative regulation of epithelial cell apoptotic process | 0.04443 |
| positive regulation of cell adhesion | 0.04565 |
| heart looping | 0.04704 |
| oligosaccharide metabolic process | 0.04704 |
| endothelial cell apoptotic process | 0.04704 |
| regulation of nitric oxide metabolic process | 0.04704 |
| regulation of cell-substrate adhesion | 0.0478 |
| kidney development | 0.04786 |
| heart morphogenesis | 0.048 |
| heart valve development | 0.04837 |
| regulation of phospholipase activity | 0.04837 |
| negative regulation of neuron apoptotic process | 0.0495 |

Supplement Table 5. Correlation analysis was conducted with 52 normal tissue samples, 539 primary and 100 metastatic PCa tumor samples (correlation coefficient of 0.5 was used).

| **gene** | **correlation coefficient** | **p-value** |
| --- | --- | --- |
| CDKN2B-AS1 | 0.483 | 1.2492E-41 |
| AKT1 | -0.398 | 1.1041E-27 |
