# Supplementary material for: The oncogenic lncRNA MIR503HG suppresses cellular senescence counteracting supraphysiological androgen treatment in prostate cancer
Source: J Exp Clin Cancer Res. 2024 Dec 16;43:321. doi: 10.1186/s13046-024-03233-2 (PMC11648305; doi:10.1186/s13046-024-03233-2)
Supplement: Supplementary file 4 — Supplementary Material 4. [file 13046_2024_3233_MOESM4_ESM.pptx]

## Slide 1
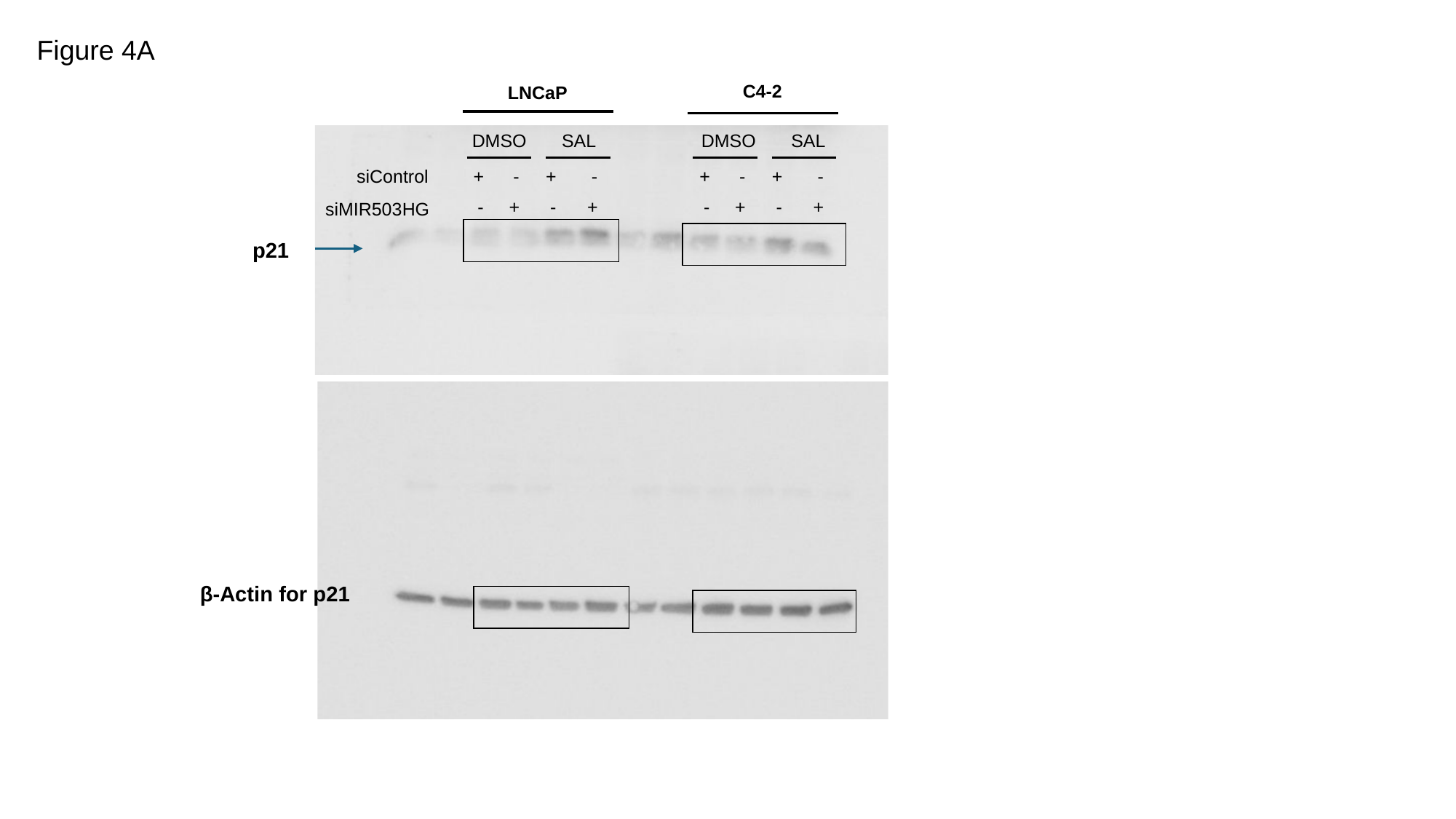

Figure 4A
C4-2
LNCaP
DMSO
SAL
DMSO
SAL
+
-
+
-
+
-
+
-
siControl
-
+
-
+
-
+
-
+
siMIR503HG
p21
β-Actin for p21

## Slide 2
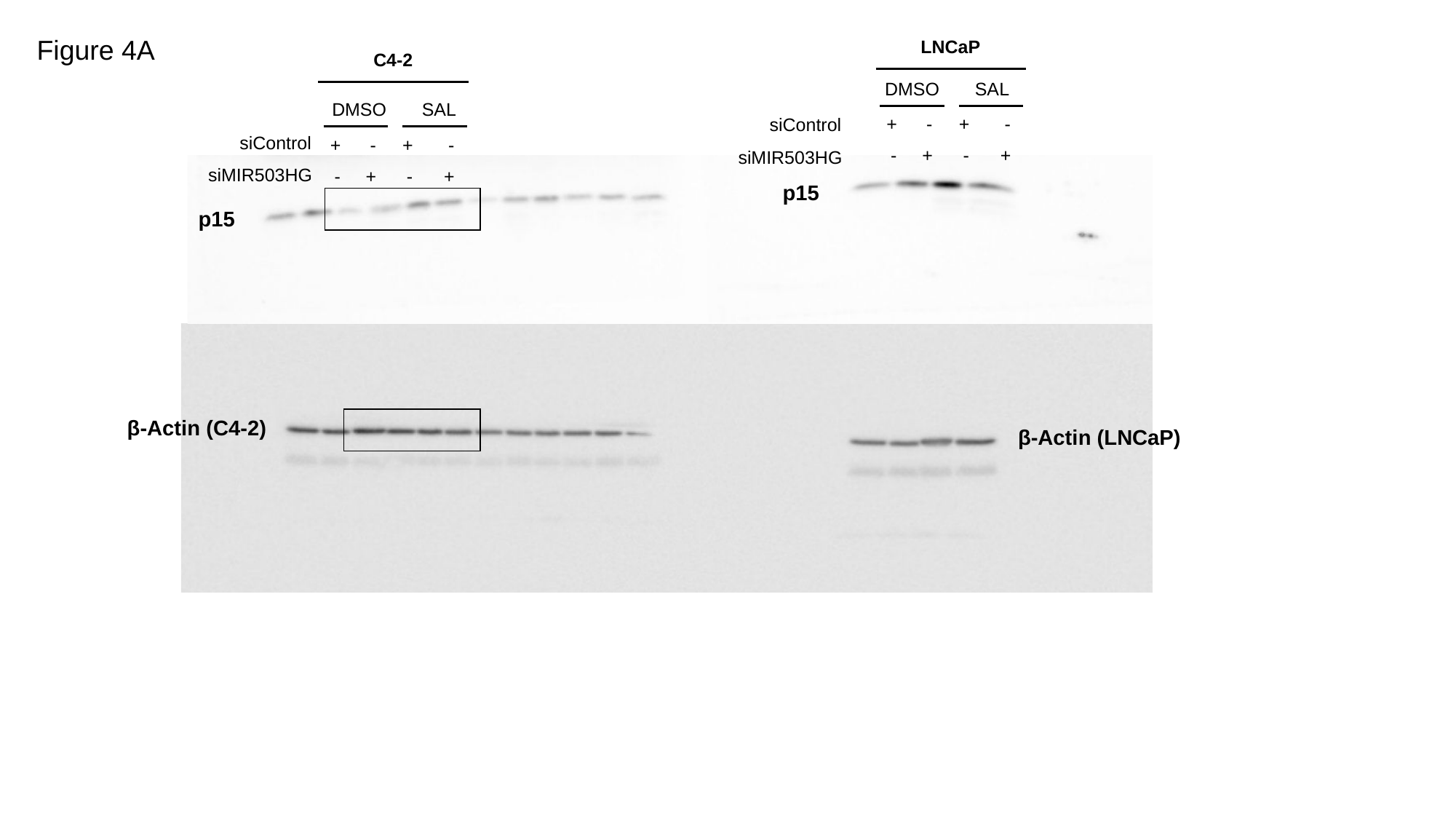

Figure 4A
LNCaP
C4-2
DMSO
SAL
DMSO
SAL
+
-
+
-
siControl
siControl
+
-
+
-
-
+
-
+
siMIR503HG
siMIR503HG
-
+
-
+
p15
p15
β-Actin (C4-2)
β-Actin (LNCaP)

## Slide 3
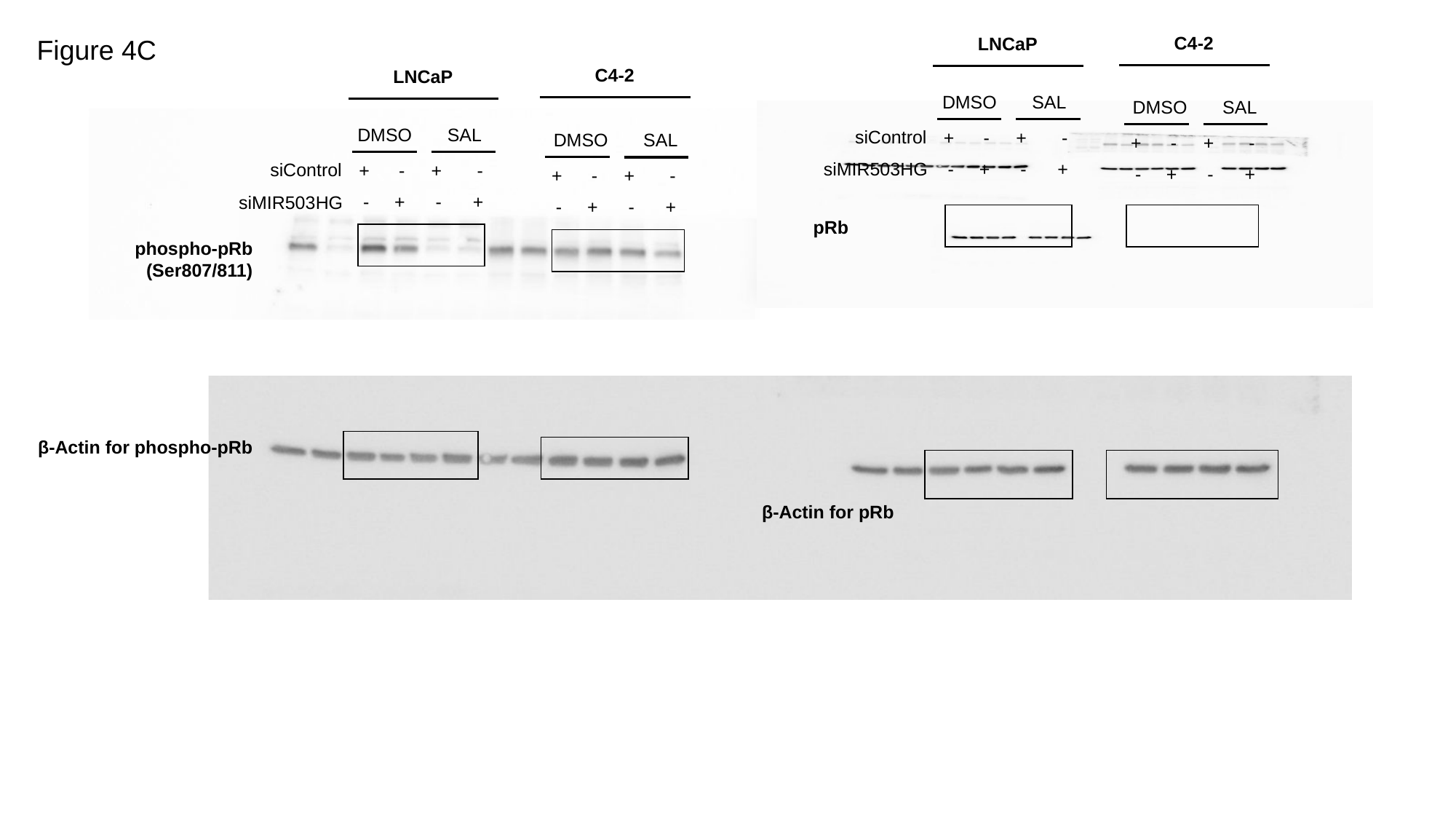

C4-2
LNCaP
Figure 4C
C4-2
LNCaP
DMSO
SAL
DMSO
SAL
DMSO
SAL
siControl
+
-
+
-
DMSO
SAL
+
-
+
-
-
+
-
+
siMIR503HG
siControl
+
-
+
-
-
+
-
+
+
-
+
-
-
+
-
+
siMIR503HG
-
+
-
+
pRb
phospho-pRb
(Ser807/811)
β-Actin for phospho-pRb
β-Actin for pRb

## Slide 4
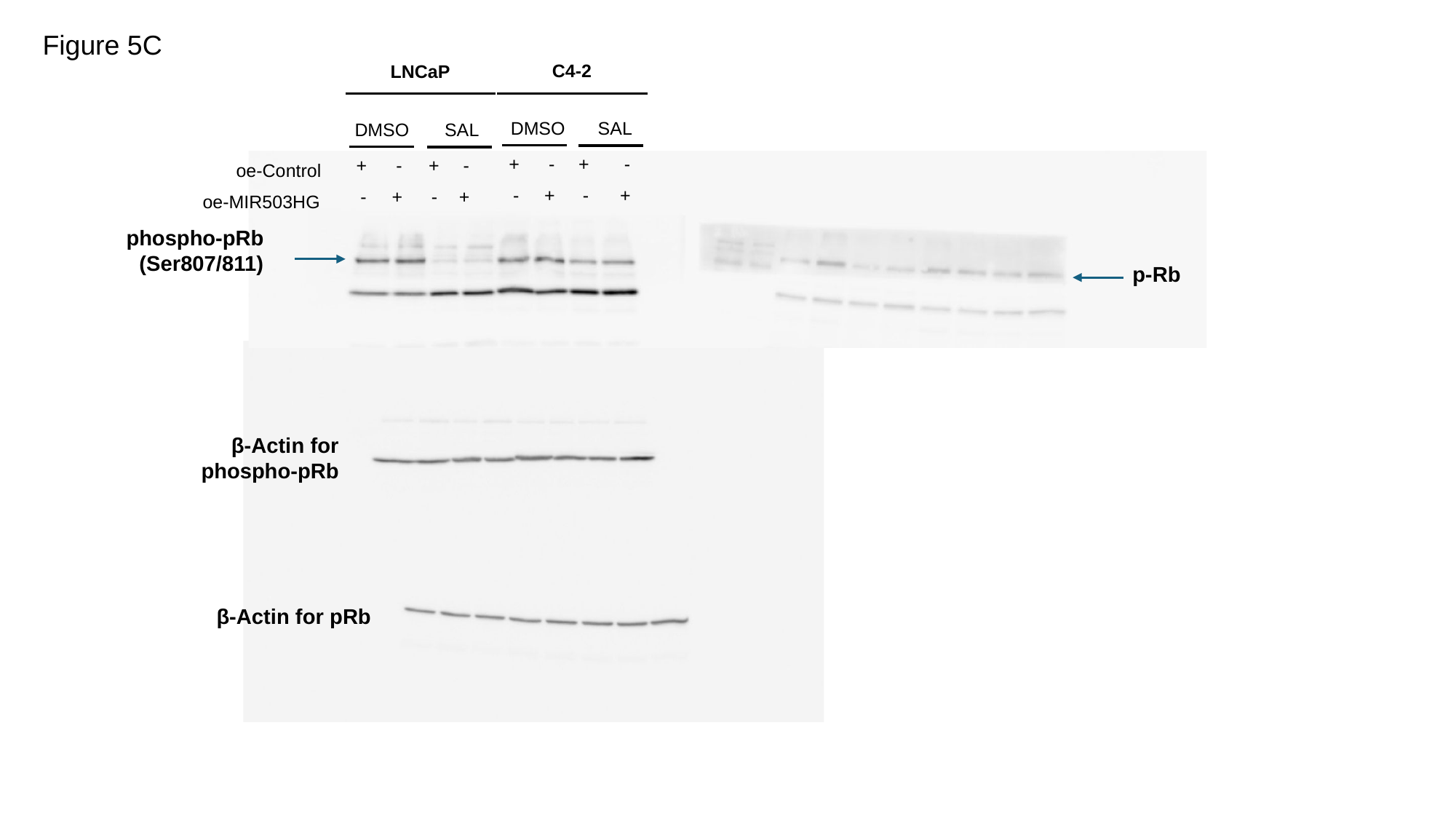

Figure 5C
C4-2
LNCaP
DMSO
SAL
DMSO
SAL
+
-
+
-
+
-
+
-
oe-Control
-
+
-
+
-
+
-
+
oe-MIR503HG
phospho-pRb (Ser807/811)
p-Rb
β-Actin for phospho-pRb
β-Actin for pRb

## Slide 5
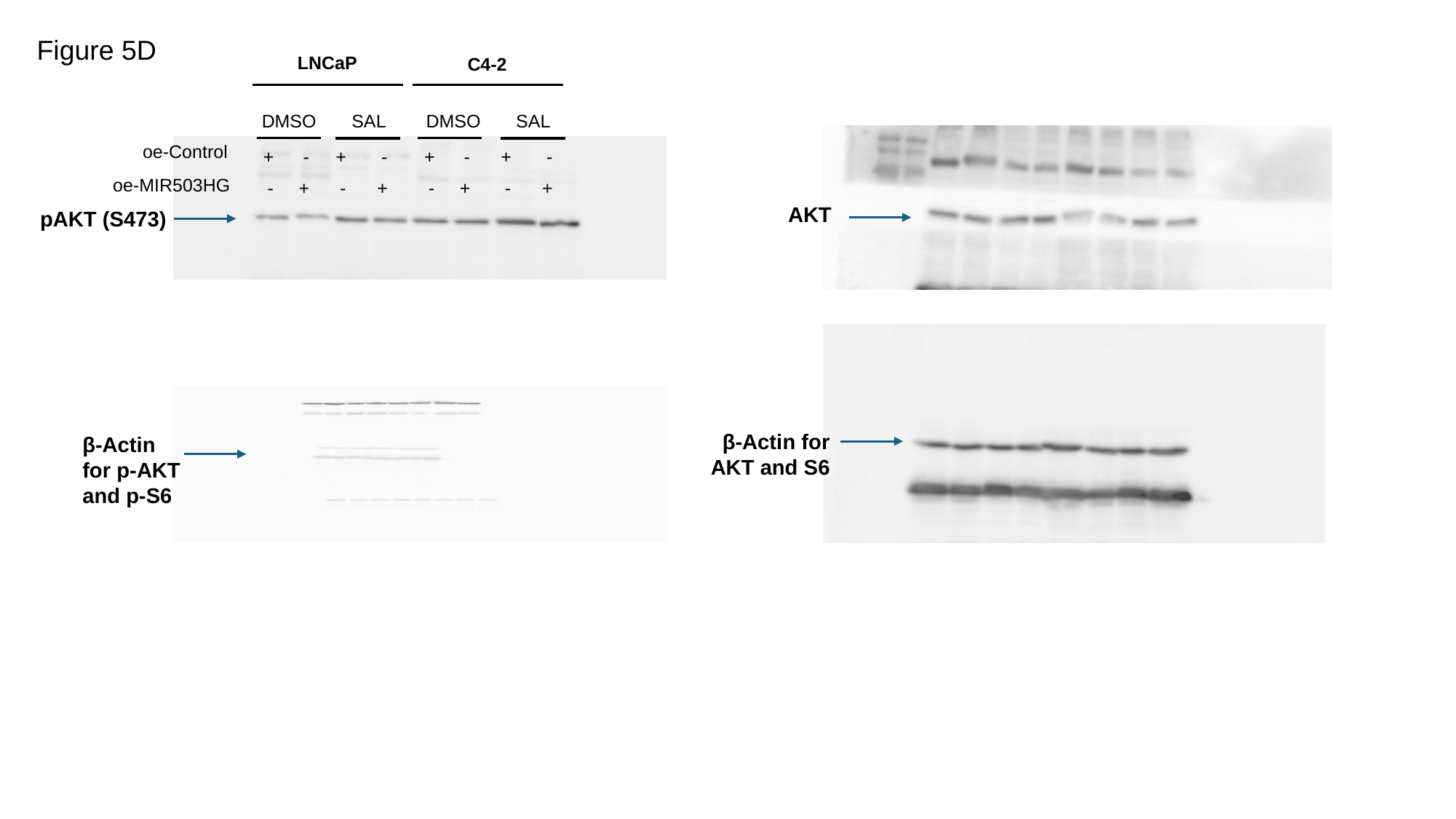

Figure 5D
LNCaP
C4-2
DMSO
SAL
DMSO
SAL
oe-Control
+
-
+
-
+
-
+
-
oe-MIR503HG
-
+
-
+
-
+
-
+
AKT
pAKT (S473)
β-Actin for AKT and S6
β-Actin
for p-AKT and p-S6

## Slide 6
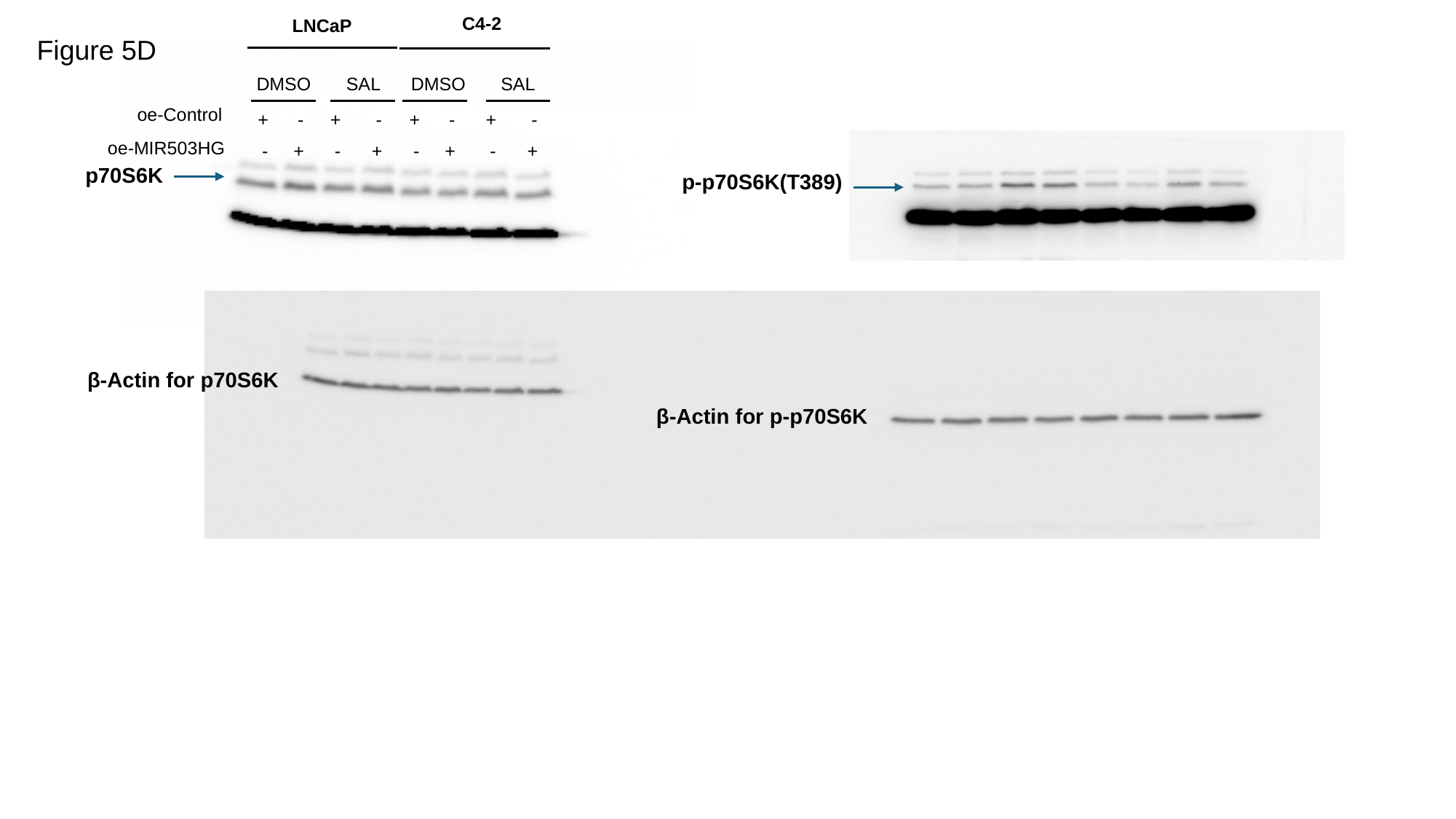

C4-2
LNCaP
Figure 5D
DMSO
SAL
DMSO
SAL
oe-Control
+
-
+
-
+
-
+
-
oe-MIR503HG
-
+
-
+
-
+
-
+
p70S6K
p-p70S6K(T389)
β-Actin for p70S6K
β-Actin for p-p70S6K

## Slide 7
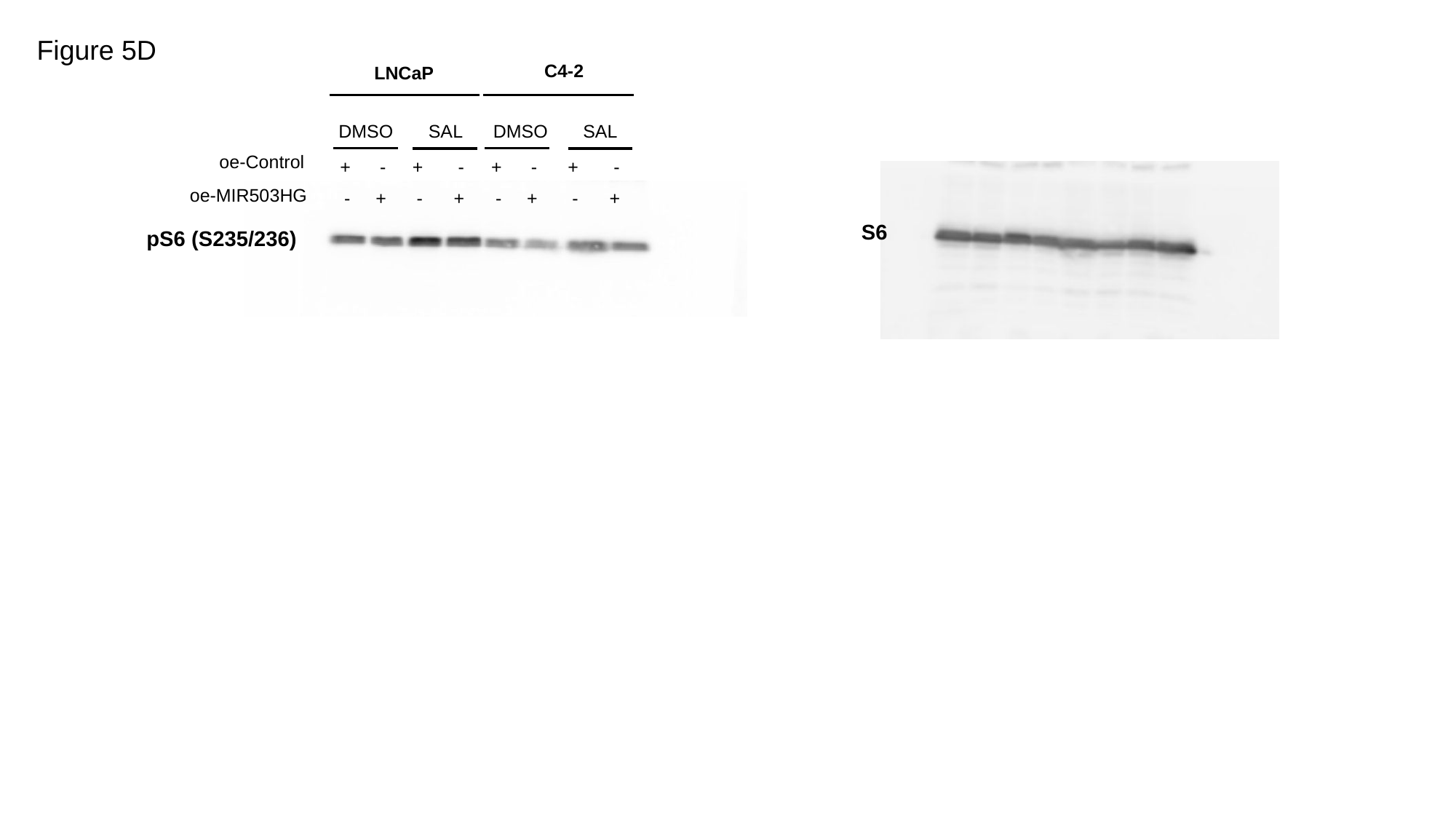

Figure 5D
C4-2
LNCaP
DMSO
SAL
DMSO
SAL
oe-Control
+
-
+
-
+
-
+
-
oe-MIR503HG
-
+
-
+
-
+
-
+
S6
pS6 (S235/236)

## Slide 8
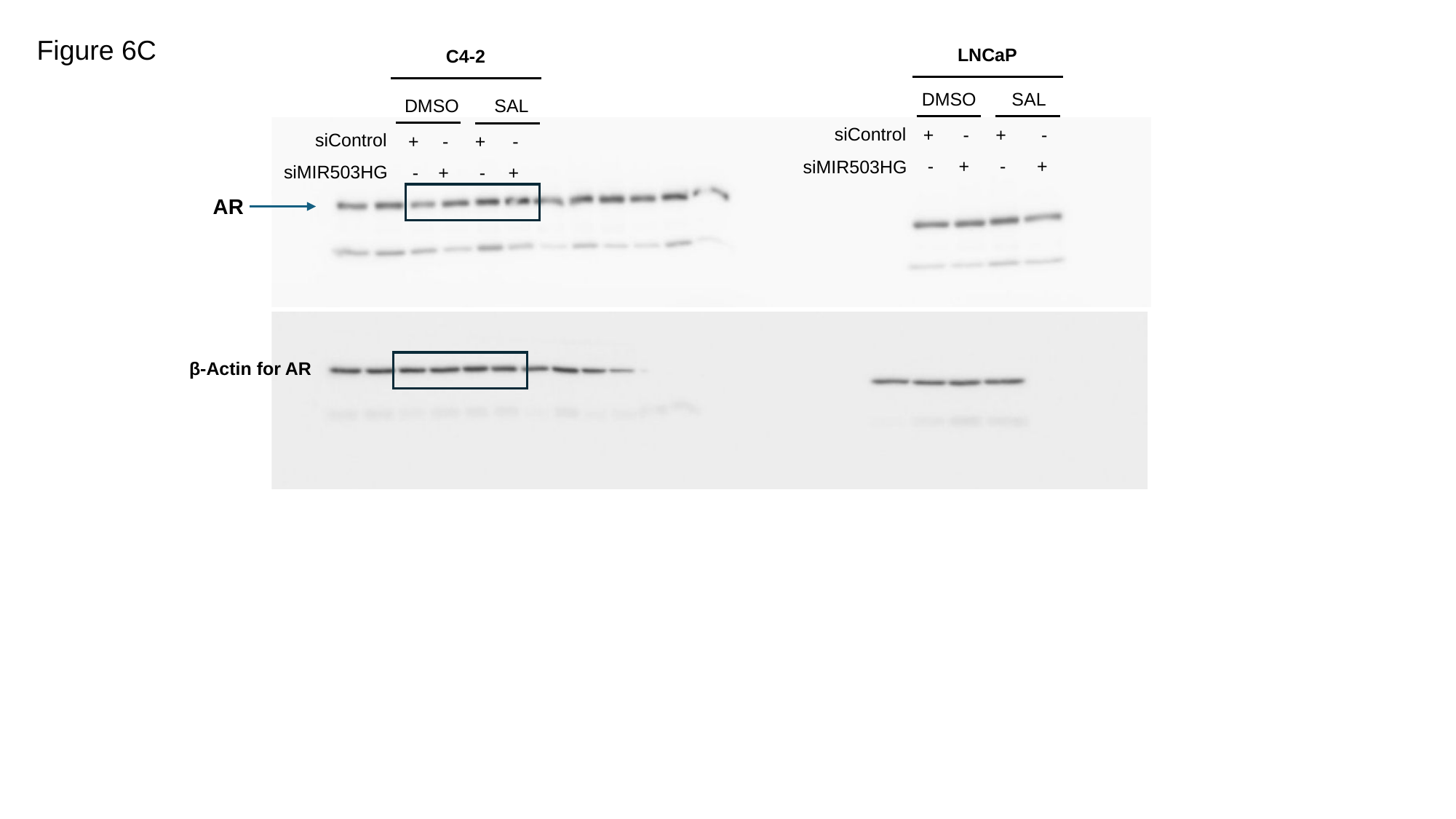

Figure 6C
LNCaP
C4-2
DMSO
SAL
DMSO
SAL
siControl
+
-
+
-
siControl
+
-
+
-
-
+
-
+
siMIR503HG
siMIR503HG
-
+
-
+
AR
β-Actin for AR

## Slide 9
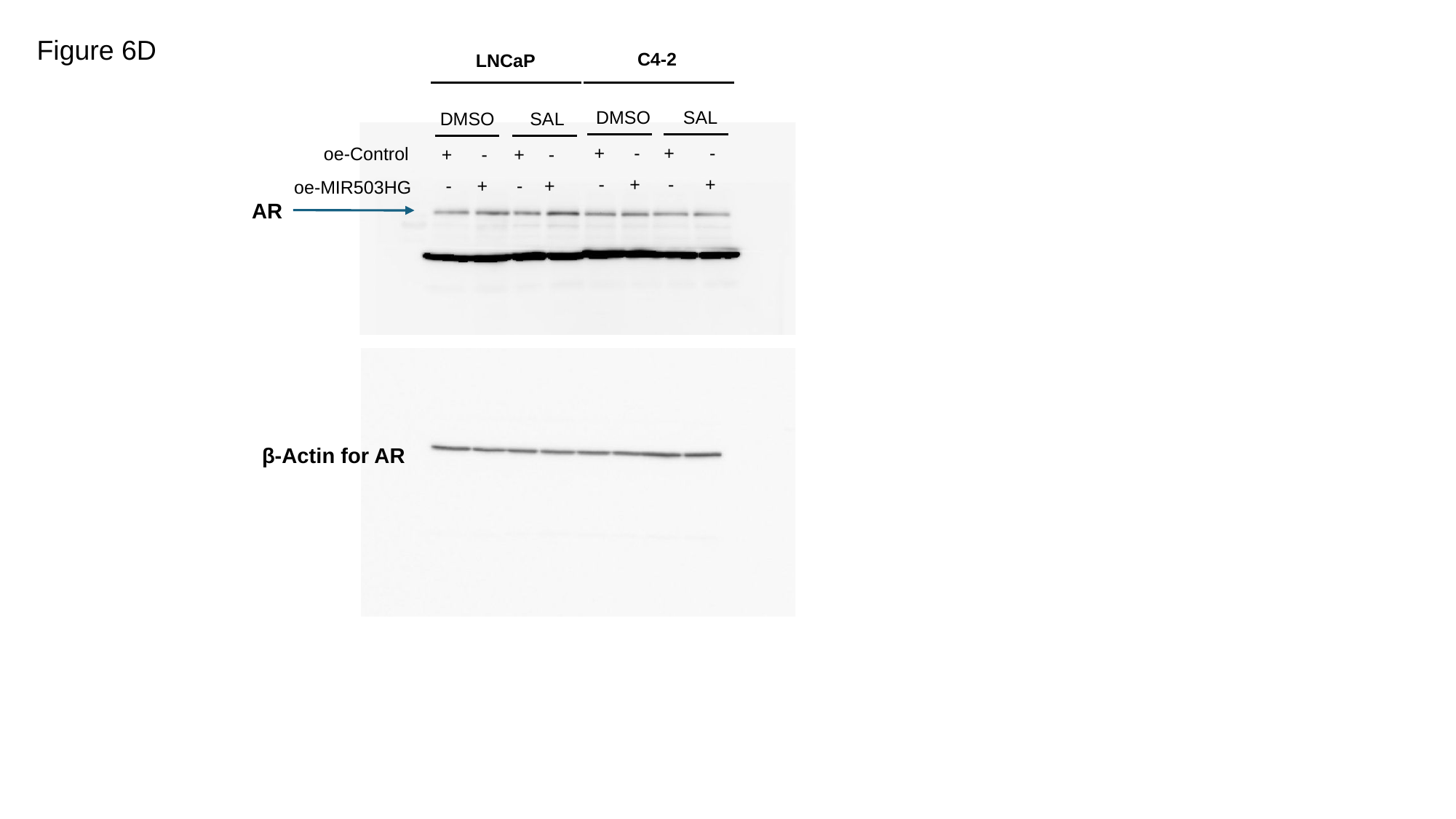

Figure 6D
C4-2
LNCaP
DMSO
SAL
DMSO
SAL
+
-
+
-
oe-Control
+
-
+
-
-
+
-
+
-
+
-
+
oe-MIR503HG
AR
β-Actin for AR
